# Supervised, structured and individualized exercise in metastatic breast cancer: a randomized controlled trial

**DOI:** 10.1038/s41591-024-03143-y

**Published:** 2024-07-25

**Authors:** Anouk E. Hiensch, Johanna Depenbusch, Martina E. Schmidt, Evelyn M. Monninkhof, Mireia Pelaez, Dorothea Clauss, Nadira Gunasekara, Philipp Zimmer, Jon Belloso, Mark Trevaskis, Helene Rundqvist, Joachim Wiskemann, Jana Müller, Maike G. Sweegers, Carlo Fremd, Renske Altena, Maciej Gorecki, Rhodé Bijlsma, Lobke van Leeuwen-Snoeks, Daan ten Bokkel Huinink, Gabe Sonke, Ainhara Lahuerta, G. Bruce Mann, Prudence A. Francis, Gary Richardson, Wolfram Malter, Elsken van der Wall, Neil K. Aaronson, Elzbieta Senkus, Ander Urruticoechea, Eva M. Zopf, Wilhelm Bloch, Martijn M. Stuiver, Yvonne Wengstrom, Karen Steindorf, Anne M. May

**Affiliations:** 1 Julius Center for Health Sciences and Primary Care, University Medical Center Utrecht, Utrecht University, Utrecht, The Netherlands; 2 German Cancer Research Center (DKFZ) and National Center for Tumor Diseases (NCT, a partnership between DKFZ and University Medical Center Heidelberg) Heidelberg, Heidelberg, Germany; 3 Gipuzkoa Cancer Unit, OSID-Onkologikoa, BioGipuzkoa, Osakidetza, San Sebastian, Spain; 4 Universidad Europea del Atlantico, Santander, Spain; 5 German Sport University Cologne, Cologne, Germany; 6 TU Dortmund University, Dortmund, Germany; 7 Mary MacKillop Institute for Health Research, Australian Catholic University, Melbourne, Victoria Australia; 8 Department of Laboratory Medicine, Karolinska Institutet and Unit of Clinical Physiology, Karolinska University Hospital, Stockholm, Sweden; 9 Heidelberg University Hospital and NCT Heidelberg (a partnership between DKFZ and University Medical Center Heidelberg), Heidelberg, Germany; 10 Netherlands Cancer Institute, Amsterdam, The Netherlands; 11 Department of Medical Oncology, National Center for Tumor Diseases (NCT), University Hospital Heidelberg, Heidelberg, Germany; 12 Divison of Gynecologic Oncology, National Center for Tumor Diseases (NCT), University Hospital Heidelberg, Heidelberg, Germany; 13 German Cancer Research Center Heidelberg (DKFZ), Heidelberg, Germany; 14 Karolinska Institutet, Unit for Nursing, Karolinska Comprehensive Cancer Center, Breast and Sarcoma Unit, Karolinska University Hospital, Solna, Sweden; 15 Greater Poland Cancer Centre, Poznan, Poland; 16 Division of Imaging and Oncology, University Medical Center Utrecht, Utrecht University, Utrecht, The Netherlands; 17 Diakonessenhuis Utrecht–Zeist–Doorn, Utrecht, The Netherlands; 18 Alexander Monro Ziekenhuis, Bilthoven, The Netherlands; 19 Royal Melbourne Hospital, Melbourne, Victoria, Australia; 20 Peter MacCallum Cancer Centre, The University of Melbourne, Victoria, Australia; 21 Cabrini Research, Cabrini Health, Malvern, Victoria Australia; 22 Center for Familial Breast and Ovarian Cancer, Faculty of Medicine and University Hospital Cologne, University of Cologne, Cologne, Germany; 23 Medical University of Gdańsk, Gdańsk, Poland

**Keywords:** Outcomes research, Breast cancer

## Abstract

Physical exercise both during and after curative cancer treatment has been shown to reduce side effects. Evidence in the metastatic cancer setting is scarce, and interventions that improve health-related quality of life (HRQOL) are much needed for patients with metastatic breast cancer (MBC). The multinational randomized controlled PREFERABLE-EFFECT trial assessed the effects of exercise on fatigue and HRQOL in patients with MBC. In total, 357 patients with MBC and a life expectancy of ≥6 months but without unstable bone metastases were recruited at eight study centers across five European countries and Australia. Participants were randomly assigned (1:1) to usual care (control group, *n* = 179) or a 9-month supervised exercise program (exercise group, *n* = 178). Intervention effects on physical fatigue (European Organization for Research and Treatment of Cancer (EORTC) Quality of Life Questionnaire (QLQ)-FA12 scale) and HRQOL (EORTC QLQ-C30 summary score) were determined by comparing the change from baseline to 3, 6 (primary timepoint) and 9 months between groups using mixed models for repeated measures, adjusted for baseline values of the outcome, line of treatment (first or second versus third or higher) and study center. Exercise resulted in significant positive effects on both primary outcomes. Physical fatigue was significantly lower (−5.3 (95% confidence interval (CI), −10.0 to −0.6), Bonferroni–Holm-adjusted *P* = 0.027; Cohen's effect size, 0.22) and HRQOL significantly higher (4.8 (95% CI, 2.2–7.4), Bonferroni–Holm-adjusted *P* = 0.0003; effect size, 0.33) in the exercise group than in the control group at 6 months. Two serious adverse events occurred (that is, fractures), but both were not related to bone metastases. These results demonstrate that supervised exercise has positive effects on physical fatigue and HRQOL in patients with MBC and should be recommended as part of supportive care.

ClinicalTrials.gov Identifier: NCT04120298.

## Abstract (web summary)

In this large interventional, randomized controlled trial, structured and individualized exercise in patients with metastatic breast cancer led to lower levels of fatigue and improved health-related quality of life.

## Main

Breast cancer is currently the most commonly diagnosed cancer worldwide. With about 2.3 million new cases in 2020, it poses a significant burden to public health^1,2^. In 2020, almost 700,000 people worldwide died from breast cancer, with advanced disease being the main cause^1^. Owing to therapeutic advances for patients with MBC, survival time has improved (it ranges from a median of 23 to 64 months, depending on tumor receptor status)^3–5^. Consequently, optimizing MBC patients’ HRQOL is a crucial aim, as indicated by 80% of the patients^6^. Nevertheless, there is no evidence of improvement in HRQOL in MBC patients over the past decade, and in certain cases, a decline has even been observed^6^. Patients often experience debilitating cancer-related and treatment-related side effects, including fatigue, decreased physical fitness, anxiety and depression, neuropathy and pain^7,8^. Of these side effects, fatigue is the most reported and burdensome symptom^9^, and has the largest negative impact on HRQOL^6^. Given that patients with metastatic disease typically receive ongoing treatment, these side effects may worsen over time. Cumulative symptom burden can be associated with treatment adjustments or discontinuation, which may negatively affect survival^10^. Therefore, interventions that can improve HRQOL by alleviating fatigue and other side effects are much needed for patients with MBC.

Recent evidence-based international guidelines recommend physical exercise both during and after curative cancer treatment to reduce side effects^11,12^. However, the guidelines acknowledge that, in the context of MBC, the evidence of the effects of exercise is scarce, and no recommendations can be provided. Two systematic reviews in patients with advanced cancer, including patients with MBC, concluded that exercise interventions are safe and feasible^13,14^, and that this is also true for patients with bone metastases^15^. The studies in these reviews suggest that exercise improves physical fitness and functioning in patients with MBC. However, insufficient data are available to draw any conclusions regarding the effects of exercise on fatigue, HRQOL and other cancer-related and treatment-related side effects^13^. Hence, a high-quality and adequately powered study is needed to assess the effects of exercise in patients with MBC.

The randomized controlled PREFERABLE-EFFECT study was designed to assess the effects of a structured and individualized 9-month exercise intervention on fatigue and HRQOL in patients with MBC. The secondary aims were to investigate the effects of exercise on other cancer-related and treatment-related side effects. Here, we report on the primary outcomes and a range of secondary outcomes of the PREFERABLE-EFFECT study.

## Results

### Patient characteristics

**Fig. 1:**
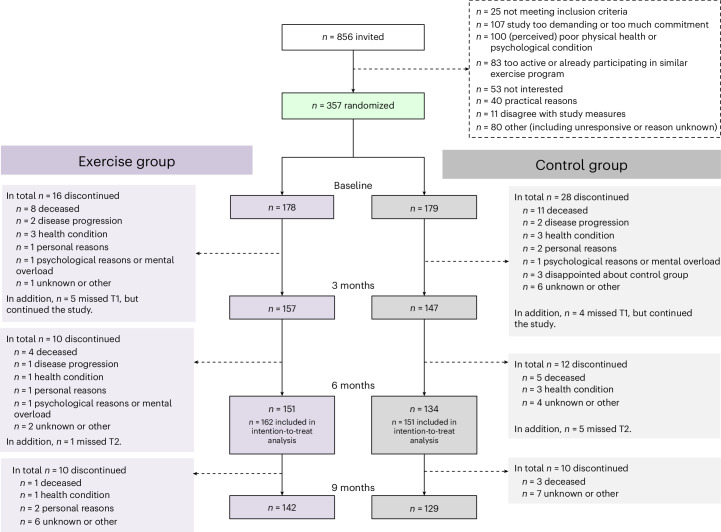
CONSORT diagram. Flow of participants through the study.

Between 8 January 2020 and 3 August 2022, a total of 856 patients were invited to take part in the PREFERABLE-EFFECT study. Of these, 357 provided consent and were enrolled (recruitment rate, 41.7%), with 178 randomized to the exercise group and 179 to the control group (Fig. 1). The overall dropout rates were 12.3% at 3 months, 18.5% at 6 months and 24.1% at 9 months. The median (interquartile range (IQR)) attendance rate for supervised exercise sessions was 77% (48–92%) (see Extended Data Table 1 for reasons for missed exercise sessions). Median compliance with the exercise protocol was 70% for the balance component, 59%–83% for the aerobic components and 63%–100% for the resistance components (Extended Data Table 2).

**Extended Data Fig. 1:**
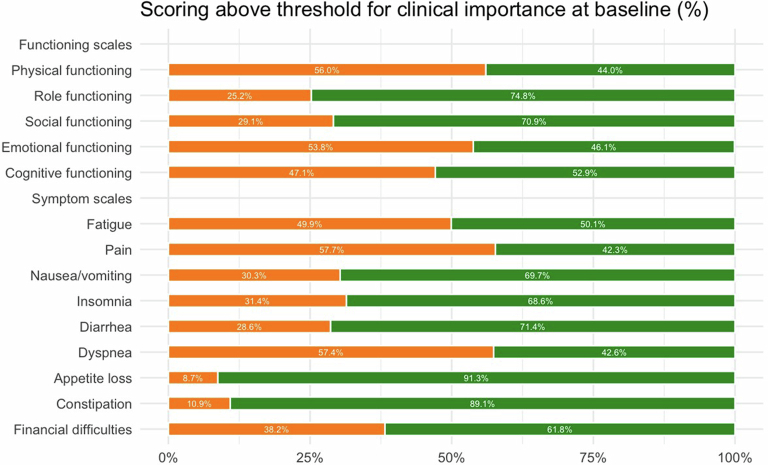
Percentage of participants scoring above (orange) or below (green) the threshold for clinical importance for the functional and symptom scales of the EORTC QLQ-C30 at baseline^16^.

The exercise and control groups had similar sociodemographic and clinical characteristics at baseline (Table 1). The mean age of the participants was 55.4 years (s.d., 11.1), and the majority were female (99.4%), were receiving first-line or second-line treatment (74.8%) and had bone metastases (67.2%). At baseline, about half of all participants reported fatigue above the threshold for clinical importance (score on the QLQ-C30 fatigue scale ≥ 39; 49.9%), and more than half reported low physical functioning (score < 83; 56.0%), pain (score ≥ 25; 57.7%) and dyspnea (score ≥ 17; 57.4%) (Extended Data Fig. 1). These thresholds were determined in a previous study based on external criteria (for example, limitations in daily living and perceived need for care), which reflect the clinical importance of a health problem^16^.

**Table 1:** Baseline characteristics of the EFFECT participants

	Exercise group (*n* = 178)	Control group (*n* = 179)
**Sociodemographic characteristics**		
**Age** **(years)**	54.9 (11.6)	55.9 (10.7)
**Sex**		
Female	177 (99.4)	178 (99.4)
Male	1 (0.6)	1 (0.6)
**Marital status**		
Married	121 (68.0)	117 (65.4)
Living alone	57 (32.0)	62 (34.6)
**Education**		
No or basic education	7 (3.9)	5 (2.8)
Middle education	35 (19.7)	40 (22.3)
Higher education	37 (20.8)	43 (42.0)
Academic education	99 (55.6)	90 (50.3)
Other	0 (0.0)	1 (0.6)
**Body mass index** **(kg** **m**^**−2**^**)**	25.9 (5.1)	26.6 (5.3)
Underweight	6 (3.4)	2 (1.1)
Normal weight	85 (47.8)	77 (43.0)
Overweight or obese	87 (48.9)	100 (55.9)
**Smoking status**		
Never smoked	93 (52.2)	92 (51.4)
Former smoker	76 (42.7)	67 (37.4)
Current smoker	9 (5.1)	20 (11.2)
**Work status**		
Employed	83 (46.6)	77 (43.0)
On sick leave	34 (41.0)	43 (55.8)
Permanently disabled	31 (17.4)	33 (18.4)
Unemployed	2 (1.1)	3 (1.7)
Retired	38 (21.3)	34 (19.0)
Home duties	12 (6.7)	14 (7.8)
Other^a^	11 (6.2)	28 (15.6)
**Physical activity (median (IQR) min per week)**		
Aerobic exercise		
Vigorous intensity	0 (0–0)	0 (0–0)
Moderate intensity	0 (0–58)	0 (0–60)
Light intensity	30 (0–150)	15 (0–132)
Resistance exercises	0 (0–0)	0 (0–0)
**Clinical characteristics**		
**Disease presentation at the time of MBC diagnosis**		
De novo stage IV	58 (32.6)	59 (33.0)
Recurrent disease	116 (65.2)	111 (62.0)
Time since MBC diagnosis (median (IQR) months)	23.1 (8.1–54.3)	22.5 (9.1–49.9)
**Line of treatment**		
First	98 (55.1)	91 (50.8)
Second	36 (20.2)	42 (23.5)
Third or higher	44 (24.7)	46 (24.6)
**Tumor subtype**		
Triple-negative	13 (7.3)	22 (12.3)
HER2-positive	42 (23.6)	41 (22.9)
HER2-negative and hormone receptor-positive	108 (60.7)	106 (59.2)
**Current treatment**		
Endocrine treatment	94 (52.8)	93 (52.0)
Targeted therapy^b^	105 (59.0)	99 (55.3)
Chemotherapy	48 (27.0)	43 (24.0)
Bone-modifying agent	83 (46.6)	83 (46.4)
**Previous cancer treatment**		
Primary surgery	120 (67.4)	120 (67.0)
Surgery of metastases	21 (11.8)	17 (9.5)
Chemotherapy	115 (64.6)	114 (64.0)
Endocrine treatment	96 (53.9)	96 (53.6)
Radiotherapy	111 (62.4)	95 (53.1)
**Location of metastases**		
Bone	116 (65.2)	124 (69.3)
Lung	49 (27.5)	46 (25.7)
Liver	67 (37.6)	57 (31.8)
Lymph node	68 (38.2)	70 (39.1)
**Comorbidities**		
0	104 (58.4)	104 (58.4)
1	37 (20.8)	33 (18.4)
>1	37 (20.8)	42 (23.5)

Continuous characteristics are presented as mean (s.d.), whereas categorical characteristics are presented as *n* (%), unless stated otherwise.

^a^Includes caregiver, working on projects when health status allows.

^b^Includes both biologic agents, such as cyclin-dependent kinase 4 (CDK4) and CDK6 inhibitors, as well as human epidermal growth factor receptor 2 (HER2)-targeted therapy.

### Primary outcomes

**Fig. 2:**
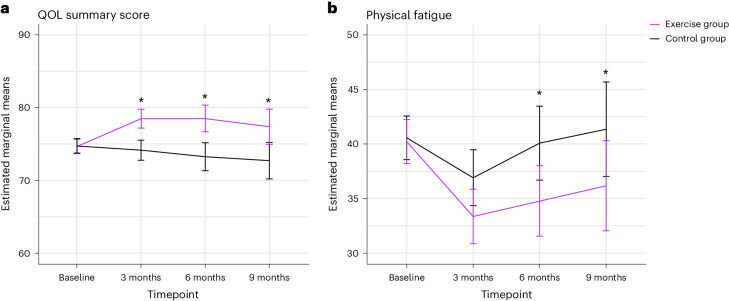
Changes in QOL summary score and physical fatigue over the 9-month intervention period in both the exercise and control groups. Changes in the QOL summary score over the 9-month intervention period (**a**) and changes in physical fatigue (**b**). Asterisk, statistically significant BGD.

The exercise group reported significantly better HRQOL than the control group at 6 months (between-group difference (BGD), 4.8 (95% CI, 2.2–7.4); Bonferroni–Holm-adjusted *P* = 0.0003; effect size (ES), 0.33). At 6 months, the exercise group also reported significantly lower physical fatigue levels compared to the control group (BGD, −5.3 (95% CI, −10.0 to −0.6); Bonferroni–Holm-adjusted *P* = 0.027; ES, 0.22) (Table 2 and Fig. 2a,b).

**Table 2:** Effects of the EFFECT exercise program on HRQOL, fatigue, physical fitness and activity outcomes

		BGDs at 3 months		BGDs at 6 months		BGDs at 9 months	
		Mean (95% CI)	ES	Mean (95% CI)	ES	Mean (95% CI)	ES
**Primary outcomes**							
Summary score (EORTC QLQ-C30)	EX vs CG	3.9 (1.5–6.3)*	0.27	4.8 (2.2–7.4)*	0.33	4.3 (1.4–7.3)*	0.30
Physical fatigue (EORTC QLQ-FA12)	EX vs CG	−3.4 (−7.8–1.0)	0.14	−5.3 (−10.0 to −0.6)*	0.22	−5.6 (−10.9 to −0.4)*	0.24
**Sensitivity analysis**							
Multiple imputation							
Summary score	EX vs CG	4.4 (1.7–7.2)*	0.31	4.8 (2.3–7.3)*	0.33	4.4 (1.7–7.2)*	0.31
Physical fatigue	EX vs CG	−3.9 (−8.2–0.3)	0.16	−5.6 (−10.1 to −1.0)*	0.24	−5.6 (−10.7 to −0.6)*	0.24
**EORTC QLQ-C30**							
Global QOL	EX vs CG	2.6 (−1.1–6.4)	0.15	4.7 (0.8–8.7)*	0.27	4.5 (0.3–8.6)*	0.25
Physical functioning	EX vs CG	3.9 (0.8–6.9)*	0.23	7.0 (3.6–10.3)*	0.42	5.9 (2.2–9.6)*	0.33
Physical functioning T-score^a^	EX vs CG	2.5 (1.3–3.8)*	0.34	3.2 (1.8–4.7)*	0.43	3.0 (1.3–4.7)*	0.40
Emotional functioning	EX vs CG	−0.2 (−4.3–3.8)	0.01	0.8 (−3.3–4.9)	0.03	0.9 (−3.6–5.3)	0.04
Role functioning	EX vs CG	5.1 (−0.2–10.5)	0.20	7.6 (2.1–13.1)*^#^	0.29	5.0 (−1.1–11.1)	0.19
Social functioning	EX vs CG	4.0 (−1.0–8.9)	0.14	5.5 (0.2–10.8)*	0.20	8.9 (3.1–14.6)*^#^	0.32
Cognitive functioning	EX vs CG	4.0 (0.4–7.5)*	0.16	3.5 (−0.3–7.2)	0.14	2.5 (−1.5–6.6)	0.10
Fatigue	EX vs CG	−5.4 (−9.8 to −1.0)*	0.22	−8.0 (−12.7 to −3.4)*^#^	0.32	−6.4 (−11.6 to −1.2)*	0.26
Pain	EX vs CG	−4.9 (−9.8 to −0.03)*	0.20	−7.1 (−12.1 to −1.9)*	0.28	−6.5 (−12.0 to −1.0)*	0.26
Nausea and vomiting	EX vs CG	−0.6 (−3.4–2.2)	0.04	−2.4 (−5.5–0.7)	0.18	−2.5 (−6.1–1.0)	0.19
Dyspnea	EX vs CG	−4.1 (−8.6–0.3)	0.16	−7.6 (−12.2 to −3.0)*	0.28	−3.9 (−9.0–1.3)	0.14
Insomnia	EX vs CG	−5.7 (−11.1 to −0.2)*	0.19	−4.7 (−10.7–1.3)	0.16	−4.4 (−10.9–2.1)	0.15
Appetite loss	EX vs CG	−1.9 (−6.8–3.1)	0.08	−3.9 (−8.7–1.0)	0.17	−3.8 (−8.9–1.2)	0.16
Constipation	EX vs CG	−4.7 (−9.3–0.04)	0.18	0.3 (−4.5–5.1)	0.01	−0.6 (−6.2–4.9)	0.03
Diarrhea	EX vs CG	−2.3 (−6.9–2.3)	0.09	−0.4 (−5.2–4.4)	0.02	−1.8 (−6.9–3.2)	0.07
Financial difficulties	EX vs CG	−1.9 (−6.1–2.3)	0.07	−1.1 (−5.6–3.4)	0.04	−1.6 (−6.4–3.2)	0.06
**EORTC QLQ-FA12**							
Emotional fatigue	EX vs CG	1.3 (−3.3–5.8)	0.05	−3.2 (−8.1–1.7)	0.13	−1.1 (−6.3–4.1)	0.05
Cognitive fatigue	EX vs CG	−1.1 (−4.6–2.3)	0.06	−2.4 (−5.7–0.9)	0.12	−1.5 (−5.0–1.9)	0.08
Interference with daily activities	EX vs CG	−1.1 (−6.9–4.6)	0.04	−5.0 (−10.8–0.8)	0.19	−4.8 (−10.8–1.1)	0.18
Social sequelae	EX vs CG	−2.6 (−7.0–1.9)	0.11	−2.4 (−7.1–2.2)	0.10	−5.1 (−10.0 to −0.1)*	0.21
**Physical fitness**							
MSEC (W)	EX vs CG	23.2 (15.8–30.6)*	0.44	24.8 (17.3–32.3)*	0.47	Not tested	Not tested
**Self-reported physical activity** (Godin–Shephard Leisure-Time Exercise Questionnaire)							
Aerobic exercise							
Vigorous intensity (min per week)	EX vs CG	23 (14–33)*	0.73	24 (15–33)*	0.77	15 (5–24)*	0.48
Moderate intensity (min per week)	EX vs CG	19 (−2–41)	0.24	2 (−20–23)	0.02	28 (7–49)*	0.35
Light intensity (min per week)	EX vs CG	−21 (−59–18)	0.13	−6 (−44–32)	0.04	−14 (−57–29)	0.08
Resistance exercise (min per week)	EX vs CG	30 (19–41)*	1.17	38 (27–49)*	1.49	16 (5–26)*	0.63
**Measured physical activity using the Fitbit Inspire HR**^**b**^							
Steps per day	EX vs CG	261 (−590–1,111)	0.05	682 (−232–1,597)	0.14	761 (−378–1,899)	0.16
Sedentary (min per day)	EX vs CG	−39 (−102–24)	0.15	−65 (−135–5)	0.24	−85 (−166 to −4)*	0.32
Lightly active (min per day)	EX vs CG	13 (−9–34)	0.15	14 (−10–37)	0.17	10 (−18–38)	0.12
Fairly active (min per day)	EX vs CG	−0.4 (−5–4)	0.03	0.6 (−4–5)	0.04	5 (−0.2–10)	0.32
Very active (min per day)	EX vs CG	2 (−2–6)	0.10	7 (2–12)*	0.36	7 (1–13)*	0.36

Models were adjusted for the baseline value of the outcome and stratification factors (that is, center and therapy line) and included participants for whom the outcome was observed at two or more timepoints. Suggested interpretation by Cohen: ES < 0.2, no difference; ES of 0.2–0.5, small difference; ES of 0.5–0.8, medium difference; ES ≥ 0.8, large difference. *Indicates significant differences (*P* < 0.05). Significant BGDs are in bold. ^#^Indicates that the BGD is larger than the MID, which has been estimated in patients with advanced breast cancer and can be interpreted as the smallest change in a HRQOL outcome that is perceived as important by a patient^28^. The following MIDs have been established and are only available to EORTC QLQ-C30 symptom and functional scales: 8 (physical functioning), 4 (role functioning), 7 (social functioning), 4 (cognitive functioning), 10 (global QOL) and 8 (fatigue). EX, exercise group; CG, control group.

^a^To assess higher levels of physical functioning, four additional items from the EORTC item bank were used. A domain-specific T-score was calculated for physical functioning using EORTC software. This T-score reflects the position of the participant relative to an age-matched and gender-matched European reference population, with 50 representing average physical functioning.

^b^These models included participants for whom valid Fitbit data (that is, wearing the Fitbit for >4 days around measurement timepoints for ≥10 h per day) could be retrieved (CG, 106; EX, 111).

### Secondary outcomes

**Fig. 3:**
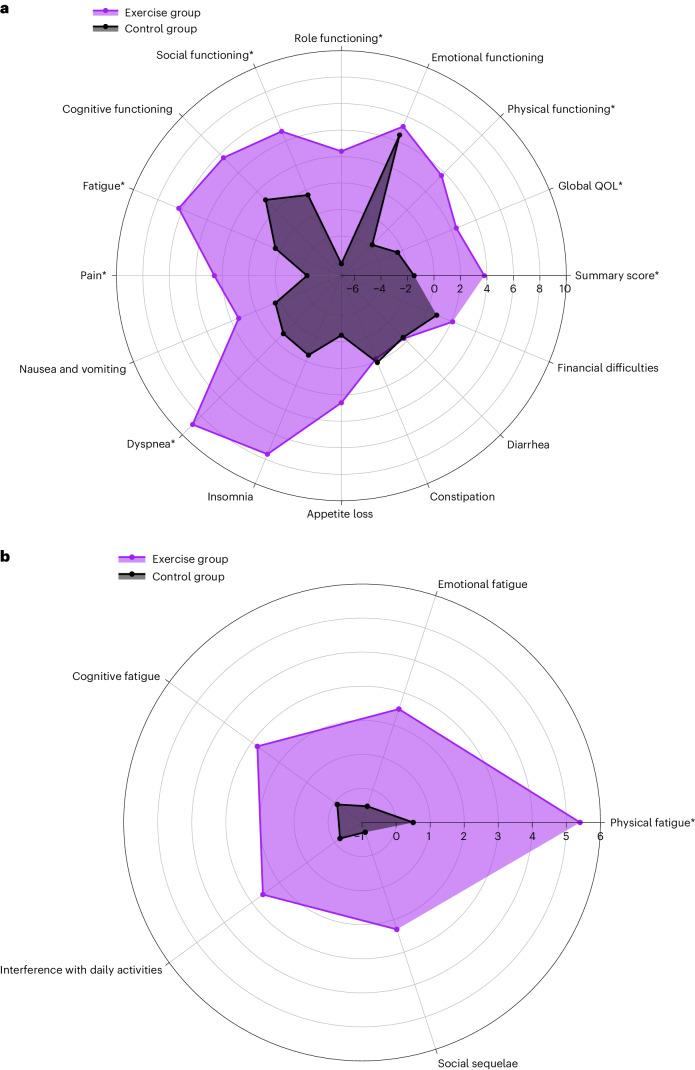
Radar plots demonstrating changes from baseline to 6 months post baseline in quality of life and fatigue scores for participants randomized to the exercise or control groups. A radar plot for all quality of life outcomes (**a**) and a radar plot for all fatigue dimensions (**b**). It should be noted that the scale of all quality of life symptom outcomes and fatigue outcomes were inverted to facilitate interpretability. An increase from baseline to 6 months post baseline now indicates an improvement for all outcomes. Asterisk, statistically significant BGD.

Alongside the observed beneficial effects of exercise at 6 months regarding the primary outcomes, the exercise group reported significantly better HRQOL than the control group at 3 months (BGD, 3.9 (95% CI, 1.5–6.3); ES, 0.27) and 9 months (BGD, 4.3 (95% CI, 1.4–7.3); ES, 0.30). At 9 months, the exercise group also reported significantly lower levels of physical fatigue than the control group (BGD, −5.6 (95% CI, −10.9 to −0.4); ES, 0.24). At 6 months, we found positive exercise effects on physical fitness (BGD, 24.8 W (95% CI, 17.3–32.3); ES, 0.47) and on numerous QLQ-C30 scales, including global QOL (BGD, 4.7 (95% CI, 0.8–8.7); ES, 0.27), physical functioning (BGD, 7.0 (95% CI, 3.6–10.3); ES, 0.42), role functioning (BGD, 7.6 (95% CI, 2.1–13.1); ES, 0.29), social functioning (BGD, 5.5 (95% CI, 0.2–10.8); ES, 0.20), pain (BGD, −7.1 (95% CI, −12.1 to −1.9); ES, 0.28) and dyspnea (BGD, −7.6 (95% CI, −12.2 to −3.0); ES, 0.28) (Table 2 and Fig. 3a). Positive exercise effects on physical functioning and pain were already observed at 3 months (BGD, 3.9 (95% CI, 0.8–6.9) and ES, 0.23 for physical functioning; BGD, −4.9 (95% CI, −9.8 to −0.03) and ES, 0.20 for pain) and remained throughout the entire intervention period up to 9 months (BGD, 5.9, (95% CI, 2.2–9.6) and ES, 0.33 for physical functioning; BGD, −6.5 (95% CI, −12.0 to −1.0) and ES, 0.26 for pain). At 3 months, the exercise group reported significantly better cognitive functioning (BGD, 4.0, (95% CI, 0.4–7.5); ES, 0.16) and less insomnia (BGD, −5.7 (95% CI, −11.1 to −0.2); ES, 0.19) than the control group; however, these BGDs were no longer significant at 6 and 9 months. No beneficial effects of the exercise intervention were found on emotional functioning or emotional fatigue (Table 2 and Fig. 3a,b). Compared to the control group, the exercise group significantly increased self-reported vigorous-intensity aerobic exercise levels at 3, 6 and 9 months (BGD, 23 min per week (95% CI, 14–33) and ES, 0.73; BGD, 24 min per week (95% CI, 15–33) and ES, 0.77; and BGD, 15 min per week (95% CI, 5–24) and ES, 0.48, respectively), as well as resistance exercise levels (BGD, 30 min week (95% CI, 19–41) and ES, 1.17; BGD, 38 min per week (95% CI, 27–49) and ES, 1.49; and 16 min per week (95% CI, 5–26) and ES, 0.63, respectively) (Table 2 and Supplementary Table 1). Similarly, compared to the control group, the exercise group significantly increased objectively measured ‘very active’ physical activity levels at 6 months (BGD, 7 min per day (95% CI, 2–12); ES, 0.36) and 9 months (BGD, 7 min per day (95% CI, 1–13); ES, 0.36), whereas sedentary time was significantly decreased at 9 months (BGD, −85 min per day, (95% CI, −166 to −4); ES, 0.32). At 9 months, the exercise group also increased self-reported moderate-intensity aerobic exercise levels (BGD, 28 min per week (95% CI, 7–49); ES, 0.35) compared to the control group. No significant BGDs were found for other physical activity levels. Within-group differences for all outcomes are shown in Extended Data Tables 3–5.

### Safety

In total, two exercise-related serious adverse events (SAEs) were reported: a wrist fracture and a sacral stress fracture. These were not related to bone metastases. Both participants continued the exercise program after an interruption and/or a modification to the program. Frequently reported adverse events (AEs) (*n* = 80), which required modifications to the exercise program, included pain (51.3%), dizziness (12.5%), muscle soreness and/or cramps (11.3%) and fatigue (8.9%).

### Exploratory outcomes

**Fig. 4:**
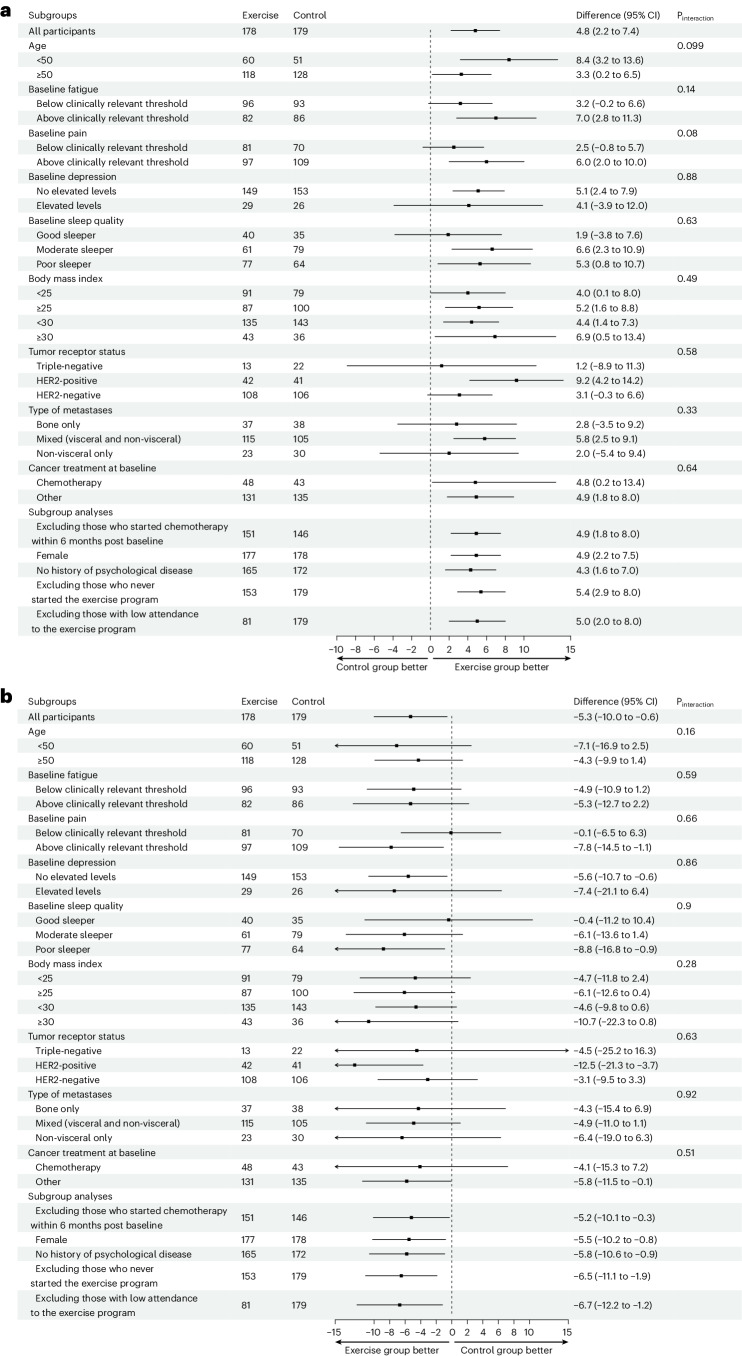
Moderators of the effects of exercise on the primary outcomes HRQOL and physical fatigue. Moderation effects are shown for the primary outcomes HRQOL (**a**) and physical fatigue (**b**), measured at 6 months (that is, the primary endpoint).

Regarding effect modification, exercise effects on HRQOL and physical fatigue did not vary significantly as a function of clinical characteristics or baseline values of physical fitness or patient-reported outcomes (PROs) (Fig. 4a,b). Greater effects on HRQOL were found for participants who were younger (BGD, 8.4 (95% CI, 3.2–13.6) for those <50 years old (31%) versus BGD, 3.3 (95% CI, 0.2–6.5) for those ≥50 years old) or who reported pain above the clinically important threshold at baseline (58%) compared to participants without pain (BGD, 6.0 (95% CI, 2.0–10.0) versus BGD, 2.5 (95% CI, −0.8–5.7).

## Sensitivity analysis

The sensitivity analysis, with multiple types of imputed data, yielded similar results; that is, the exercise group reported significantly better HRQOL (BGD, 4.8, (95% CI, 2.3–7.3); ES, 0.33) and lower physical fatigue levels than the control group at 6 months (BGD, −5.6 (95% CI, −10.1 to −1.0), ES, 0.24).

## Discussion

In the PREFERABLE-EFFECT study, we have demonstrated that patients with MBC were able to participate in a 9-month supervised exercise program that resulted in improvements in fatigue and HRQOL at 6 months, which were maintained for up to 9 months. In addition, beneficial effects on clinically relevant outcomes such as physical functioning, role functioning, physical fitness, dyspnea and pain were observed.

Current American College of Sports Medicine (ACSM)^11^ and American Society of Clinical Oncology^12^ guidelines recommend exercise during and after curative cancer treatment and report a lack of studies in the metastatic setting. Our results may contribute to future updates to these guidelines. Furthermore, our results may facilitate the inclusion of more detailed exercise recommendations in the current European School of Oncology–European Society for Medical Oncology international consensus guidelines for advanced breast cancer^17^. Currently, the guidelines do not include specific exercise recommendations for patients with metastatic disease owing to a lack of supporting empirical data. So far, only six randomized controlled exercise trials have been conducted in patients with MBC, with sample sizes ranging from 14 to 101 patients^18–23^. These were primarily feasibility studies and were not powered to detect statistically significant differences in fatigue or HRQOL between groups. Only two randomized controlled trials (*n* = 38 and *n* = 40) reported statistically significant positive effects of exercise on fatigue and HRQOL compared to a control group^21,23^. Recently, an adequately powered (*n* = 313) randomized controlled trial investigated the effects of a remotely supervised, 16-week home-based exercise program on HRQOL in patients with advanced pancreatic ductal adenocarcinoma^24^. Although the exercise program in that study was considered safe and feasible, no statistically significant beneficial effects of exercise on various dimensions of HRQOL, including global health status, fatigue and physical functioning, were found. The lack of an effect might be attributed, in part, to the underlying disease, which is characterized by a more aggressive clinical disease course and often involves multiple nutrition-related symptoms requiring a different type of behavioral intervention beyond exercise only. In addition, the exercise program differed from our EFFECT exercise program in several ways, including the intensity of the program (low-to-moderate intensity versus moderate-to-high intensity), the duration of the intervention (16 weeks versus 9 months) and the setting (home-based versus hospital-based or community-based).

Current guidelines for cancer survivors recommend exercise programs of at least 12 weeks in duration during or after curative cancer treatment to improve HRQOL and fatigue^11^. In the present study, we decided to offer a 9-month exercise program, as patients with metastatic disease typically undergo continuous treatment, often leading to an exacerbation of side effects over time, and experience disease progression, leading to treatment changes and physical deterioration. This might explain why we only observed beneficial effects after 6 months for some outcomes such as physical fatigue, role functioning and social functioning. Based on this observation, we recommend exercise programs of a longer duration for patients with metastatic disease. We also reasoned that a longer intervention would improve the self-efficacy of patients, enabling them to shift from a supervised setting to an unsupervised setting. Therefore, after 6 months, we substituted one supervised session with an unsupervised session and investigated the sustainability of effects at 9 months. Furthermore, we propose that best practice should include supervision by a qualified exercise professional with expertise in the cancer setting, and preferably with experience in working with patients with metastatic cancer. At least initially, in-person supervision is preferred to ensure safety. In addition, given the complexities of the disease and its possible complications, we recommend that patients receive medical approval from their treating physician before engaging in exercise, including a risk evaluation to assess the chance of a skeletal complication arising from exercise. Information provided by the physician can help to further tailor the exercise program to accommodate the patient’s condition and ensure safety. For example, certain exercises may not be suitable for patients with bone metastases in specific locations. The International Bone Metastases Exercise Working Group advises that exercises should be avoided or adjusted based on the location of the bone metastases. Furthermore, this working group recommends that exercise prescriptions for patients with bone metastases should follow the general exercise guidelines for cancer survivors, with a focus on postural alignment, controlled movement and technique, while taking the location and clinical presentation of the bone metastases into account. This, in fact, was the procedure that was followed in our intervention protocol, which can also be applied in clinical practice^25^.

In this study, the attendance rate of 77% and the compliance rates ranging from 59 to 100% show that the patients were highly motivated and able to complete the different components of the exercise program. In previous trials, adherence to an exercise program after a cancer diagnosis ranged from 62 to 78% (ref. ^26^), which underscores the high acceptability of the supervised EFFECT exercise program. Further, the number of reported SAEs and/or AEs (*n* = 2) is comparable to what has been reported in previous studies in patients with bone metastases, and the SAEs in our study were judged to be unrelated to the bone metastases^15^. In these studies, it was concluded that exercise appeared to be safe in this patient population, but predefined safety analyses are not yet available. Despite uncertainties among healthcare professionals about recommending exercise to individuals with metastatic disease^27–29^, our findings indicate that a supervised exercise regimen like the EFFECT intervention is well tolerated by patients with MBC with stable bone metastases.

In the current study, we found statistically significant effects of exercise on several HRQOL outcomes, including fatigue. The corresponding ESs were small to moderate. These ESs are comparable to those observed in exercise–oncology trials in the curative setting^30,31^, which serve as the basis for the current exercise guidelines^11,12^. It has been argued that HRQOL outcomes in cancer studies should be interpreted based on their clinical relevance instead of their statistical significance. For this reason, minimally important differences (MIDs) have been estimated in patients with advanced breast cancer, indicating the smallest change in a HRQOL outcome that is perceived as important by a patient^32^. Although MIDs are not available for our primary outcomes (that is, the summary QLQ-C30 score and the QLQ-FA12 physical fatigue score), they are available for other HRQOL outcomes assessed in our trial. The BGDs observed in our study for fatigue, social functioning and role functioning were larger than the published MIDs. This underscores the clinical relevance of our findings. Interestingly, we did not observe any effects of exercise on emotional functioning or emotional fatigue. Based on other studies, additional interventions, such as cognitive behavioral therapy, may be needed to improve these outcomes^33,34^.

Our results indicate that the exercise intervention not only had a positive effect on physical fatigue and HRQOL but also on pain and dyspnea. This suggests that exercise may also be used as a supportive treatment for these symptoms. Indeed, recent observational data also showed that cancer survivors who are physically active experience less pain compared to those who are less active^35^. In our multinational PREFERABLE-PERSPECTIVE survey study (*n* = 420), patients with MBC frequently mentioned fatigue and pain as a barrier to start or continue exercising, and some respondents even expected exercise to worsen these symptoms^36^. The current results could therefore be used for educational and motivational purposes in helping patients with MBC to overcome exercise barriers.

Considering the acknowledged clinical thresholds of EORTC-QLQ measures, the majority of our participants reported clinically low levels of physical functioning and high levels of fatigue, pain and dyspnea at baseline^16^. In comparison to reference values of European MBC patients, our study participants reported better physical functioning but higher levels of pain, dyspnea and fatigue^9^. This indicates that patients who took part in our study were in need of an intervention to alleviate these symptoms.

This multinational randomized controlled trial is the first adequately powered study investigating the effectiveness of exercise on HRQOL and fatigue in patients with MBC. Further strengths of the study are the long duration of the exercise program and the program's supervision in different clinical settings by different exercise professionals, which resembles clinical practice in the participating countries and thereby increases the generalizability of the findings.

Our study also has some limitations. We allowed patients to enter the study at any time during their treatment, which resulted in a heterogeneous sample. However, this also increases the generalizability of our results. Conversely, we did not have detailed information on patients who declined to participate in the trial, which might hamper the generalizability of our study results. The recruitment rate was around 40%. Owing to self-selection by the patient and/or selection by the treating physician, patients who were enrolled might have had certain characteristics (for example, completed higher education) that made them more prone to participate, whereas patients in greatest need of an exercise intervention (that is, with higher fatigue levels and/or more comorbidities) might have been less likely to participate. We were also unable to blind participants to their respective study arm. This may have motivated patients in the control arm to voluntarily increase their physical activity levels, especially as all patients had received general advice on physical activity and a fitness tracker. This could have resulted in an underestimation of the intervention effect. However, based on a self-report questionnaire, we did not observe an increase in time spent on resistance exercises and vigorous-intensity aerobic exercises in the control group. It may also have led to patients in the intervention group to be more motivated during physical tests at follow-up assessments or to report more positively on PROs, which would lead to overestimation of the intervention effect. Assessment of measured physical activity was limited to aggregated data, as detailed analyses using raw Fitbit data were beyond the scope of the current analyses.

Overall, this large multinational trial demonstrated significant beneficial effects of a supervised exercise intervention offered during oncological treatment on MBC patients’ HRQOL, fatigue and other clinically relevant outcomes. Based on our findings, we recommend that supervised resistance and aerobic exercise be included as an integral part of supportive care for patients with MBC.

## Methods

### Study design and participants

The PREFERABLE-EFFECT study design and methods have been published previously, and the full protocol is provided in the Supplementary information^37^. In brief, this multinational randomized controlled trial was undertaken at eight hospitals and study centers in Germany, the Netherlands, Poland, Spain, Sweden and Australia.

Eligible patients were 18 years of age or older, diagnosed with stage IV breast cancer, had an Eastern Cooperative Oncology Group performance status of ≤2, and were able and willing to participate in the exercise program and wear an activity tracker. Exclusion criteria were unstable bone metastases as determined by the local treating physician; untreated symptomatic brain metastases; estimated life expectancy of <6 months; serious active infection; excessive physical activity (>210 min per week of moderate-intensity to vigorous-intensity exercise) or current participation in an exercise training program comparable to the EFFECT exercise program; severe neurologic or cardiac impairment according to the ACSM criteria^38^; uncontrolled severe respiratory insufficiency or dependency on oxygen supplementation at rest or during exercise; uncontrolled severe pain; any other contraindications for exercise; any circumstances that would impede adherence to study requirements or the ability to give informed consent; or pregnancy. Patients were enrolled regardless of sex, which was collected according to the identity information provided by the patients. Patients were recruited by their clinical care or study teams or through social media (for example, national patient organizations). Medical eligibility criteria were assessed by a physician at the treating hospital.

### Ethics statement

The study was conducted in accordance with standards of good clinical practice and the Declaration of Helsinki. The study was approved by the institutional review board of the University Medical Center Utrecht, the Netherlands (19-524/M), and by the local ethical review boards of all participating institutions. The study was registered with ClinicalTrials.gov on 9 October 2019 (NCT04120298). All patients provided written informed consent before enrollment.

### Randomization and blinding

Patients who met the eligibility criteria and provided informed consent were randomly assigned (1:1), after completion of the baseline measurements, to participate in a 9-month structured and individualized exercise program in addition to usual care (exercise group) or to receive general physical activity advice in addition to usual care, but no structured exercise program (control group). All participants received an activity tracker. Randomization was performed centrally using a blocked computer-generated sequence and was stratified by study center and therapy line (first-line or second-line vs. third-line treatment or a later line of treatment). Owing to the nature of the intervention, participants, local clinicians and study nurses, and investigators were not blinded to group assignment after randomization.

### Procedures

A 9-month structured and individualized exercise program was offered to participants randomized to the exercise group. Details of the exercise program have been published elsewhere^37^. In brief, the exercise program included supervised, multimodal exercise sessions of 1 h, two times per week for the first 6 months. For the last 3 months, one supervised session was replaced by one unsupervised session. Supervision was performed by qualified exercise professionals (for example, physiotherapists and exercise physiologists) in a community-based or hospital-based fitness center, or a physical therapy practice close to the participants’ home address. In addition to the in-person supervised exercise sessions, we offered live remote exercise sessions to participants using videoconferencing software (Zoom) if training facilities were closed owing to local COVID-19 regulations or if previously enrolled participants felt unsafe exercising at a local training facility because of the COVID-19 threat.

The multimodal exercise program consisted of resistance, aerobic and balance exercises (Extended Data Table 6). Resistance exercise intensity was individualized using 12-repetition maximum muscle strength testing. For participants with bone metastases, 12-repetition maximum testing was not performed for exercises that loaded the parts of the skeleton with bone metastases (see Extended Data Table 7). During the exercise sessions, resistance exercises that loaded the affected region were either omitted or performed according to the ‘start low (that is, low weight and more repetitions), go slow (that is, gradual increase)’ principle^25^, depending on patient characteristics and the experience of the involved exercise professional. Aerobic exercise intensity was tailored to the participants’ fitness levels using the maximal short exercise capacity (MSEC) and estimated peak power output (*W*_peak_) with the steep ramp test at baseline. The intensity of both the aerobic and resistance exercises gradually increased during the exercise program; however, the intensity was continuously adjusted, depending on the health status of the participant and the participant's perceived exertion.

In addition to the supervised exercise program, participants were encouraged to be physically active for at least 30 min per day on all remaining days of the week. To support this, participants received an activity tracker (that is, Fitbit Inspire HR) and an exercise app specifically designed for the PREFERABLE-EFFECT study. The app included exercises that participants learned during the supervised exercise program and that could be performed at home. All exercises were illustrated with simple animations and contained clear instructions (see Supplementary Fig. 1 for screenshots of the app). The exercise app was also used to support the unsupervised sessions during the last 3 months of the intervention period.

Participants randomized to the control group received care as usual, supplemented with written information on the current physical activity guidelines (that is, 150 min of aerobic exercise and resistance exercise two to three times per week). They were advised to avoid inactivity and to be as physically active as their health status allowed^11^. They also received an activity tracker and an explanation of the basic functions of the tracker. The control group did not receive a structured exercise intervention, as this is not yet part of routine care.

All participants visited the study center for measurements at baseline, and at 3 and 6 months post baseline. This included the assessment of functional performance and physical fitness. At all visits as well as at 9 months post baseline, PROs were assessed using online questionnaires. Participants were asked to complete them without conferring with others. For participants undergoing intravenous chemotherapy, the measurements took place at least 3 days after chemotherapy administration. PROs, including HRQOL and fatigue, were assessed using the EORTC QLQ-C30 and the EORTC QLQ-FA12, respectively^39,40^. The QLQ-C30 is a 30-item questionnaire, including a global HRQOL score, five functional scales (physical, role, emotional, cognitive and social), three symptom scales (fatigue, nausea and vomiting, and pain) and six single items (dyspnea, insomnia, appetite loss, constipation, diarrhea and financial difficulties). A summary HRQOL score can be calculated using 13 subscales, excluding the global QOL and financial difficulties items^41^. The QLQ-FA12 is a 12-item questionnaire that assesses different dimensions of fatigue (physical, emotional, cognitive and total fatigue). For both EORTC questionnaires, scores range from 0 to 100. For the summary score, global QOL score and functional scales, higher scores indicate a higher HRQOL or a higher function, whereas for symptom scales, higher scores indicate a greater symptom burden. To assess higher levels of physical functioning, four items from the EORTC questionnaire item bank were added to the physical function scale (see Supplementary Table 2). Subsequently, a domain-specific T-score was calculated for physical functioning using EORTC software. This T-score reflects the score of the participant relative to an age-matched and a gender-matched European reference population, with 50 representing average physical functioning.

Self-reported physical activity levels were assessed using a modified version of the Godin–Shephard Leisure-Time Exercise Questionnaire^42,43^. The Godin questionnaire is a four-item questionnaire that includes questions about the average frequency and duration of mild-, moderate- and vigorous-intensity aerobic exercise and resistance exercise in bouts of at least 10 min performed during leisure time in a typical week. In addition, the Fitbit Inspire HR was used to measure daily step count and minutes of physical activity (that is, minutes per day being sedentary or lightly, fairly or very active, as classified by the FitBit software), throughout the study period. For Fitbit data, only data were used for participants who had >4 valid wear days (defined as ≥10 h of activity registration) around the measurement timepoints (that is, baseline and 3, 6 and 9 months post baseline).

As a measure of physical fitness, the MSEC was assessed with the steep ramp test using a cycle ergometer^44^. After 3 min of unloaded cycling, the test started at 25 W and was increased by 2.5 W s^−1^ or 25 W per 10 s, depending on the available settings of the cycle ergometer used, until exhaustion. Participants were instructed to cycle between 70 and 90 r.p.m. The test ended when the cycling cadence dropped below 60 r.p.m. or when the participant experienced pain or discomfort. After termination, the participant was asked to continue cycling at an easy cadence and with minimal load to promote recovery. The outcome was recorded as the highest achieved output in W and is referred to as the MSEC. From the MSEC, peak power output (*W*_peak_) was estimated using a regression equation^45^. Before physical fitness testing, resting heart rate and blood pressure were measured for safety reasons.

Body weight and height were measured in light clothing without shoes. Demographic and clinical data were extracted from questionnaires and medical records, respectively. Adherence to the supervised exercise program was recorded by the exercise professional in a case report form. Safety was assessed by the reporting of AEs and SAEs related to exercise or physical fitness testing. Participants in both groups were asked by the study personnel about exercise-related and physical fitness testing-related AEs and SAEs in a standardized manner during all follow-up visits. In addition, for participants allocated to the exercise group, the exercise professionals assessed any potential exercise-related AEs and SAEs that had occurred since the previous exercise session or during the current session and recorded this on standardized training documentation forms.

### Adherence

Adherence to the supervised exercise program was measured in terms of attendance and compliance. Attendance rates were computed as the number of supervised exercise sessions attended divided by the number of sessions offered. Compliance rates were calculated as the number of supervised exercise sessions in which participants performed all prescribed balance, resistance and aerobic exercises, divided by the number of sessions prescribed.

### Outcomes

The study had two primary outcomes: HRQOL and cancer-related physical fatigue, which were assessed using the summary score of the QLQ-C30 and the physical fatigue dimension of the QLQ-FA12, respectively. We assessed the primary outcomes at the fully supervised intervention period (that is, at 6 months) and defined the period from 6 to 9 months as the maintenance period.

Secondary outcomes reported in this paper include the primary outcomes assessed at 3 and 9 months, as well as a range of other variables: the QLQ-C30 global QOL score, and all other QLQ-C30 function and symptom scales and single items, all other QLQ-FA12 fatigue dimensions, self-reported and measured physical activity, and the MSEC.

The study included pre-planned modifier analyses for the following covariates: age (<50 vs. ≥50 years), baseline fatigue levels (QLQ-C30 fatigue scale score of <39 vs. ≥39)^16^, baseline depression levels (PHQ-4 depression subscore of <3 vs. ≥3), history of psychological disorders (any report vs. none), baseline insomnia (PSQI global score of 0–4, 5–8 or ≥9), baseline body mass index (<25 vs. ≥25 and <30 vs. ≥30), baseline fitness level (MSEC, continuous), type of therapy (chemotherapy vs. other), type of metastasis at baseline (bone only vs. mixed (visceral and non-visceral) vs. non-visceral only) and primary tumor receptor status (triple-negative vs. human epidermal growth factor receptor 2 (HER2)-positive vs. HER2-negative and hormone receptor-positive). In addition, the following subgroup analyses were prespecified: female patients only, all patients excluding those who never started the exercise program or dropped out within a month, all patients excluding those who did not adhere to the exercise program (that is, attendance of <80% of scheduled exercise sessions), all patients excluding those who began chemotherapy (intravenous or oral) between baseline and 6 months post baseline. A subgroup analysis based on baseline pain levels (QLQ-C30 pain scale score of <25 vs. ≥25)^16^ was not prespecified but became of interest during the study.

### Statistical analysis

An improvement in either or both primary outcomes in the exercise group from baseline to 6 months post baseline relative to the control group was of primary relevance. Based on a pooled analysis of six randomized controlled exercise trials in patients with breast cancer receiving adjuvant treatment, we anticipated an ES of 0.35 (ref. ^46^). With *n* = 139 patients per group (*n* = 278 in total), a mean standardized ES of at least 0.35 could be detected with a power of at least 78% or 82% at a nominal two-sided significance level of 2.5% for each outcome separately using an analysis of covariance adjusted for baseline values of the outcome variable, assuming a correlation between pre-invervention and post-intervention levels of *ρ* = 0.3 or *ρ* = 0.4, respectively^47^. To account for a potential drop-out rate of approximately 20%, the target sample size was 350 participants (*n* = 175 per study arm).

A statistical analysis plan was written before the analysis was performed and included in the study protocol. Descriptive statistics were used to characterize the study population at baseline. Questionnaire scores were calculated according to published scoring manuals. All primary analyses were performed according to the intention-to-treat principle. For the primary outcomes, linear mixed-effects models were used to assess exercise effects on physical fatigue and HRQOL separately while taking the hierarchical structure of the data into account. Models were adjusted for the baseline value of the outcome and stratification factors (that is, center and therapy line) and included participants for whom the outcome was observed at two or more timepoints. Models with different covariance structures were compared on the basis of Akaike’s information criterion. Modeling assumptions were examined and met. The same approach was used for the analysis of secondary outcomes.

Cohen’s standardized ESs were calculated by dividing the adjusted BGD of the 3-month, 6-month and 9-month post-intervention means by the pooled standard deviation at baseline. For the primary outcome, a two-tailed Bonferroni–Holm-adjusted *P* value was calculated to indicate statistical significance. For all secondary outcomes, ESs and 95% CIs are reported without *P* values. These confidence intervals are intended to express precision of the effect estimate and should not be used to infer statistical significance, as they do not account for multiple comparisons.

Prespecified intervention effect modifiers were individually added to the model as a covariate main effect and interaction effect with group allocation. Covariates that appeared to be intervention effect modifiers (*P*_interaction_ ≤ 0.10) gave rise to subgroup analyses. Prespecified subgroup analyses were performed, irrespective of interaction effects, to assess whether the intervention effect was consistent across subgroups. All modifier and subgroup analyses were treated as exploratory.

Missing values of the primary outcome variables as well as all other PROs were considered as missing at random and dealt with using linear mixed-effects models. A sensitivity analysis, using multiple imputation (*m* = 100, R package ‘MICE’)^48^, was carried out to explore potential bias and demonstrate the robustness of our results.

All statistical analyses were performed using R v4.2.2.

### Reporting summary

Further information on research design is available in the Nature Portfolio Reporting Summary linked to this article.

## Online content

Any methods, additional references, Nature Portfolio reporting summaries, source data, extended data, supplementary information, acknowledgements, peer review information; details of author contributions and competing interests; and statements of data and code availability are available at 10.1038/s41591-024-03143-y.

## Supplementary information

Supplementary Information Supplementary Information (that is, study protocol), Supplementary Fig. 1 and Supplementary Tables 1 and 2.

Reporting Summary

## Figures and Tables

**Extended Data Table 1:**
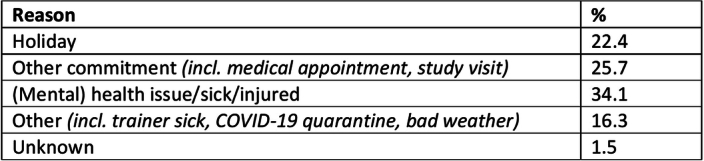
Reasons for missed supervised exercise sessions

**Extended Data Table 2:**
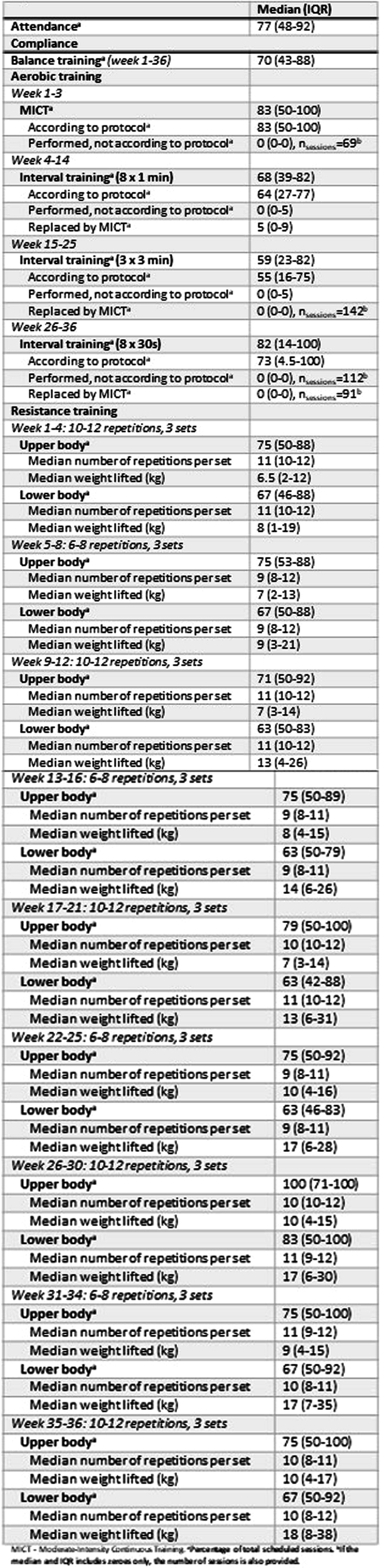
Attendance of and compliance with the supervised sessions of the EFFECT exercise program

**Extended Data Table 3:**
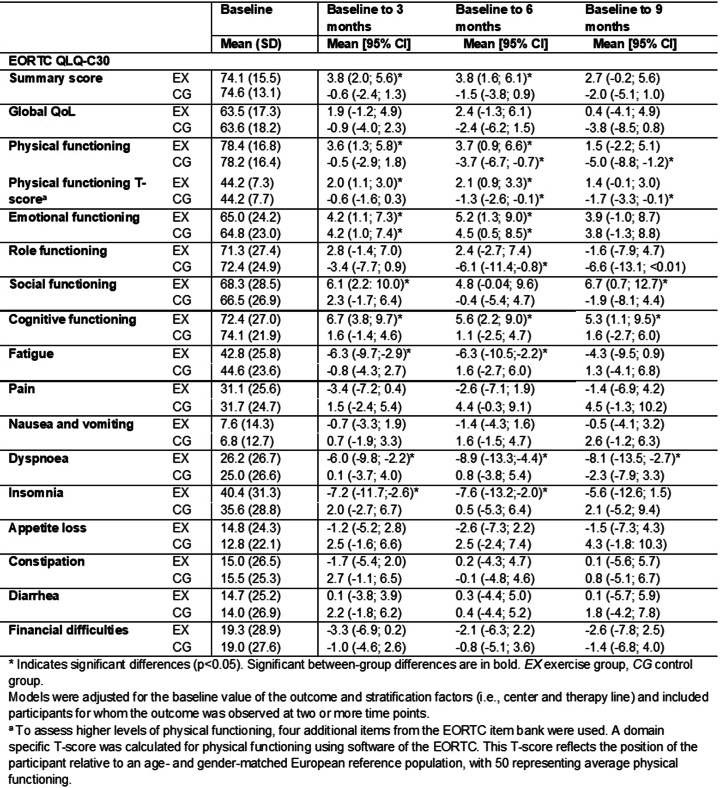
Within-group changes in HRQoL functional and symptoms scores during the 9-month intervention period

**Extended Data Table 4:**
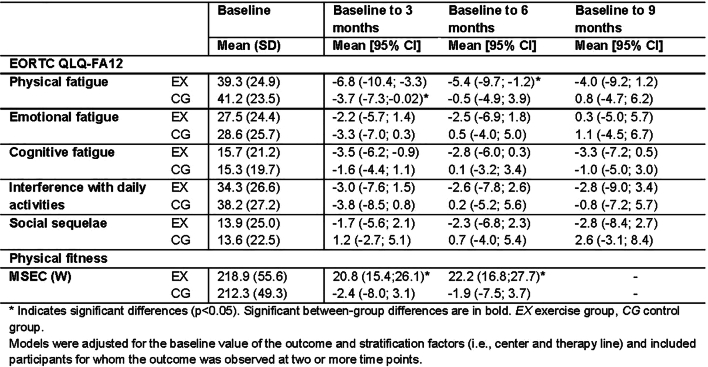
Within-group changes in fatigue and physical fitness scores during the 9-month intervention period

**Extended Data Table 5:**
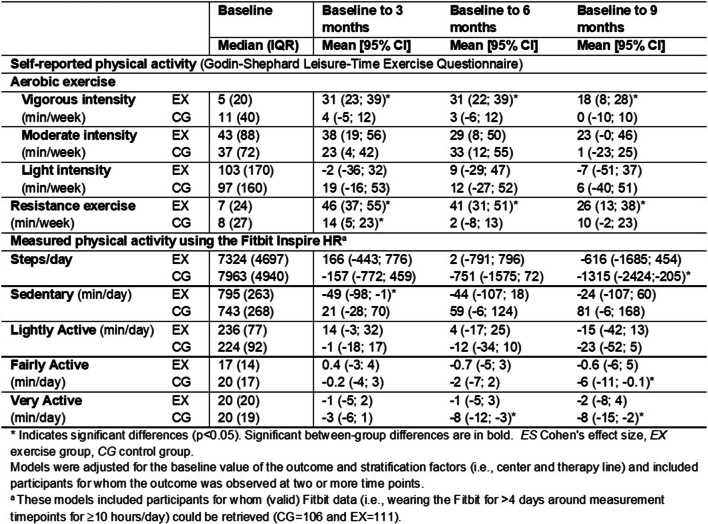
Within-group changes in self-reported and measured physical activity levels during the 9-month intervention period

**Extended Data Table 6:**
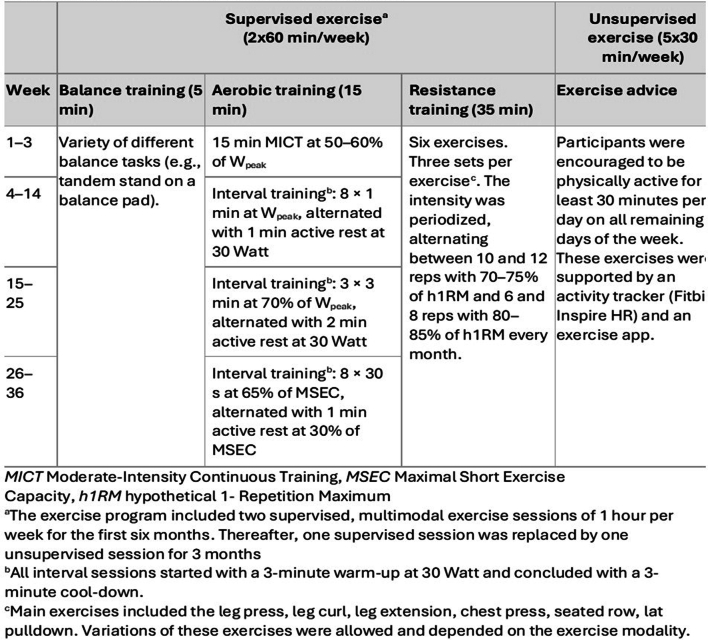
The EFFECT exercise program

**Extended Data Table 7:**
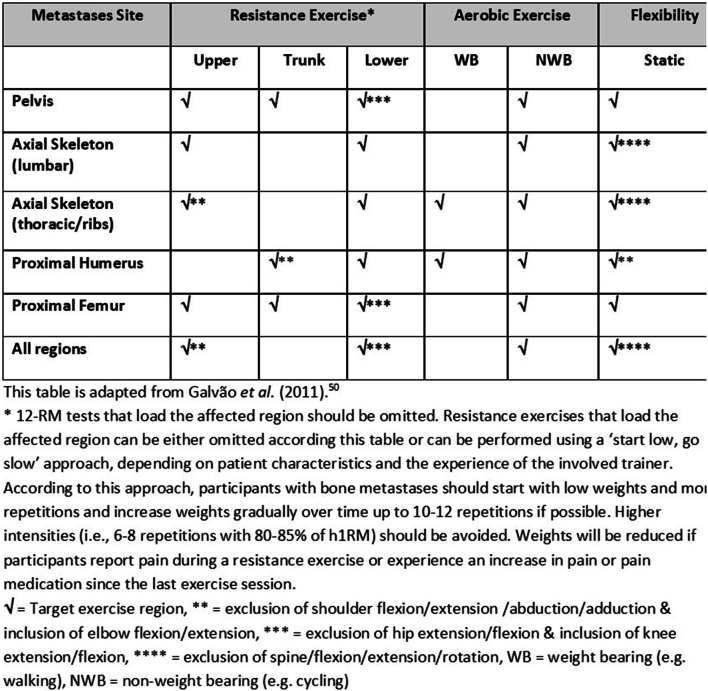
Adaptations to the prescribed exercise program based on location of bone metastases

## Data Availability

The data that support the findings of this study are not yet openly available owing to reasons of confidentiality. Researchers can request current data from the corresponding author (a.m.may@umcutrecht.nl). Pseudonymized data (including data dictionaries) will be made available through the Digital Research Environment, which is a trusted digital research environment that can be accessed at https://mydre.org. This will be carried out after the review and approval of a methodologically sound proposal by the General Assembly of PREFERABLE, with a signed data access agreement, which is in line with Ethics Committee requirements (The Ethics Committee of University Medical Center Utrecht, The Netherlands). Requests will be processed within 6 weeks. These files will be available from the date of publication until the date stated in the approved request. Once the PREFERABLE project has been fully completed, the database will be anonymized and shared using DataverseNL. The study protocol is available as an open access publication (10.1186/s13063-022-06556-7)^37^.

## Code availability

The statistical coding is available on GitHub at https://github.com/AnoukHiensch/PREFERABLE-EFFECT.git^49^.
