# Supplementary material for: Supervised, structured and individualized exercise in metastatic breast cancer: a randomized controlled trial
Source: Nat Med. 2024 Jul 25;30(10):2957–66. doi: 10.1038/s41591-024-03143-y (PMC11485212; doi:10.1038/s41591-024-03143-y)
Supplement: Supplementary file 1 — Supplementary Information (that is, study protocol), Supplementary Fig. 1 and Supplementary Tables 1 and 2. [file 41591_2024_3143_MOESM1_ESM.pdf]

nature medicine

Supplementary information

https://doi.org/10.1038/s41591-024-03143-y

Supervised, structured and individualized exercise in metastatic breast cancer: a randomized controlled trial

In the format provided by the
authors and unedited

Supplementary Information – Study Protocol EFFECT:

Effects of structured and individualized exercise in patients with metastatic breast cancer on fatigue and quality of life

**Short title**	**EFFECT**
**Version**	**4.0**
**Date**	**25 May 2021**
**Coordinating investigator/project leader**	**Prof. Dr. A.M May**<br>*On behalf of:*<br>University Medical Center Utrecht<br>Universiteitsweg 100 Str. 6.131<br>3508 GA Utrecht<br>The Netherlands<br>Tel: 0031(0)88 755 11 32<br>Email: A.M.May@umcutrecht.nl
**Local PIs**	Prof. Dr. A.M May<br>University Medical Center Utrecht, Julius Center,<br>Universiteitsweg 100 Str. 6.131, 3508 GA Utrecht,<br>The Netherlands<br><br>Prof. Dr. K. Steindorf<br>German Cancer Research Center (DKFZ)<br>Im Neuenheimer Feld 581, 69120 Heidelberg<br>Germany<br><br>Prof. Dr. med. Wilhelm Bloch<br>German Sport University Cologne)<br>Am Sportpark Müngersdorf 650933 Cologne, Germany<br><br>Dr. E. M. Zopf<br>Australian Catholic University, Mary MacKillop Institute for Health Research,Level 5, 215 Spring Street, Melbourne VIC 3000<br>Australia

	Prof. Yvonne Wengström<br>Karolinska Institutet, Dep of Neurobiology and Care Sciences, Div of Nursing, 23 300, 141 83 Huddinge<br>Sweden<br><br>Dr. M.M. Stuiver<br>Center for Quality of Life, Netherlands Cancer Institute<br>Plesmanlaan 121, 1066 CX Amsterdam<br>The Netherlands<br><br>Dr. Elżbieta Senkus-Konefka<br>Department of Oncology and Radiotherapy<br>Medical University of Gdańsk<br>Smoluchowskiego 17, 80-214 Gdańsk<br>Poland<br><br>Dr. Ander Urruticoechea Ribate<br>Fundación Onkologikoa Fundazioa<br>Paseo Dr. Begiristain 121, 20014, San Sebastian<br>Spain
**Subsidising party**	European Union (H2020 grant)<br>NHMRC (for Australian site)
**Contract Research Organisation**	Julius Clinical<br>Broederplein 41-43<br>3703 CD Zeist<br>The Netherlands
**Independent physician***	Joeke Tijink-Felderhof, Radiotherapist<br>University Medical Center Utrecht, Department of Radiotherapy<br>Phone: 088-7558800<br>J.M.Felderhof@umcutrecht.nl
**Laboratory sites**	Karolinska Institutet<br>Science for Life Laboratory<br>Box 281, 171 77 Stockholm

**Individual, knowledgeable in the field, independent of the study, who can be contacted by Dutch participants for study related questions. This individual is required by Dutch clinical trial regulations.*

OVERVIEW

**Protocol title**	The effect of structured and individualized exercise in patients with metastatic breast cancer on fatigue and quality of life
**Short title**	EFFECT
**Sponsor**	University Medical Center Utrecht<br>Heidelberglaan 100, 3584 CX Utrecht, The Netherlands
**Version**	Protocol version 4.0
**Date**	25May2021

The study is funded by the European Commission Research & Innovation Horizon 2020 program under grant agreement No. 825677

**Changes from previous version**	
Version 2.0 to 2.1	Administrative changes to adjust typing mistakes and adjustments in order to clarify the actual data collected (adjusted table 2 and appendix 2) as well as the visit time in 10.4 updated with daily practise information. Update of the correct PI.
Version 2.1 to 3.0	Administrative changes to adjust typing mistakes and adjustments in order to clarify data.<br>PI change in Australia.<br>• EORTC questionnaire will be used with additional physical function questions.<br>• Four measurements of the Short Physical Performance Battery are replaced by the short FAB scale.<br>• Adjustment of BIA measurement preparation.<br>• Possibility to (continue) exercise at home under strict conditions from the beginning of the intervention period or after a period of supervised exercise training.<br>• Collecting tracking data via the fitbit and PREFERABLE app for the entire study period instead of an intermediate period.
Version 3.0 to 4.0	Addition of the " Satisfaction with exercise intervention" questionnaire at M3 as some participants are not able to complete the M6 questionnaire, due to discontinuing the study pre-maturely for several reasons among progression of disease Addition of International Consultation on Incontinence Questionnaire Urinary Incontinence Short Form as add-on in several centers.<br>Adjustment of physical fitness testing: Measuring lower extremety strength with an isokinetic evaluation and training

	machine (Isomed 2000®) as an alternative to using a leg press machine<br>Adjustment of possibility to conduct supervised exercise sessions at home: Offering live online exercise sessions that are supervised by a physiotherapist, exercise physiologist or fitness instructor.<br>Adverse Event section update to clarify text<br>Update monitoring to remote monitoring if applicable

SYNOPSIS

**Title**	Effects of structured and individualized exercise in patients with metastatic breast cancer on fatigue and quality of life
**Short title**	**EFFECT**
**Introduction**	There is ample evidence supporting the positive impact of exercise on adverse effects of adjuvant cancer treatment. In the curative setting, exercise has shown positive results in patients undergoing breast cancer treatment in terms of reducing fatigue, but also in improving psychological symptoms, such as depression and low self-esteem. For metastatic breast cancer, the guidelines state that there is limited evidence on the potential effects of exercise and their recommendations are based on studies performed in the curative setting. Given the lack of alternatives for improving fatigue and the pressing unmet need to improve the quality of life of advanced cancer patients, exercise represents a promising and desirable intervention. Currently, the effect of exercise on metastatic breast cancer has not been extensively studied, even though the benefits are evident in the curative setting. We hypothesize that a structured and individualised exercise program will diminish levels of physical fatigue and/or the overall HRQoL-related symptom burden in patients with metastatic breast cancer.
**Study design**	The EFFECT study is a multicentre, randomised controlled trial. The intervention group will participate in a 9-month exercise intervention. The exercise program will start with a 6-month period, where patients participate in a supervised multimodal exercise program twice a week supplemented with unsupervised exercises. The multimodal exercise program comprises aerobic-, resistance- and balance components. After completing the initial six-month period, one supervised session will be replaced by one unsupervised session until month nine. Unsupervised exercises will be supported by an activity tracker (Fitbit) and an exercise App specifically designed for the EFFECT trial. Patients randomized to the control group will also receive an activity tracker (like the intervention group). We will advice control patients to avoid inactivity and be as physically active as current abilities and conditions allow, with the aim to progress towards being physically active for 150min/week in line with the current exercise guidelines.

~~**Planned Number of participants**~~	~~In total, 350 patients will be recruited.~~
**Primary Objective**	The aim is to assess the effects of a structured and individualised exercise intervention in patients with metastatic breast cancer (stage IV) on cancer-related physical fatigue, Health-Related Quality of Life (HRQoL), and other disease and treatment-related side effects at six months.
**Primary Endpoint**	Primary endpoints of the EFFECT study assessed at six months follow up are:<br>1) Cancer-related physical fatigue (EORTC QLQ-FA12)<br>2) Health-related Quality of Life (EORTC QLQ-C30 Summary Score)
**Secondary Endpoints**	Secondary outcomes are defined as:<br>• Separate HRQoL domains (EORTC QLQ-C30 function and symptom scores)<br>• Breast cancer specific symptoms (EORTC QLQ-BR45)<br>• Emotional, cognitive, and total fatigue scores (EORTC QLQ-FA12)<br>• Anxiety, depression (PHQ-4)<br>• Sleep (PSQI)<br>• Pain (BPI, painDETECT, PCS)<br>• Treatment-related toxicities grade≥3 (Common Toxicity Criteria for adverse events (CTCAE)<br>• Physical fitness/performance (Steep ramp test; endurance test; 5-times sit to stand test; short version of the Fullerton Advanced Balance (FAB) scale; handgrip- and leg strength test; and in some centers also Cardiopulmonary exercise testing (CPET))<br>• Body composition (Bio impedance, anthropometrics; and in some centers also DEXA)<br>• Physical activity levels (questionnaire / activity tracker)<br>• Profiling of circulating white blood cell populations and biomarkers (e.g., systemic inflammation, growth factors, blood/brain barrier modulators)<br>• Cost-effectiveness (iPCQ/iMCQ)/EQ-5D<br>• Quality of working life (add-on in several centers)
**Exploratory endpoints**	• Overall and breast-cancer specific survival (medical records/cancer registry)<br>• Progression-free survival (medical records/cancer registry)<br>• Maximal isokinetic and isometric leg strength, muscle thickness of m. rectus femoris (RF) and m. vastus lateralis (VL) (add-on in one center)<br>• Urinary incontinence

	~~Patients will be followed for disease progression and survival for five~~ years beyond the 9-month study period.
**Safety endpoints**	• Adverse events potentially related to the exercise intervention
**Inclusion Criteria**	In order to be eligible to participate in this study, a subject must meet all of the following criteria:<br>• Age ≥ 18 years<br>• Diagnosis of breast cancer stage IV<br>• ECOG (Eastern Cooperative Oncology Group scale) performance status ≤ 2<br>• Able and willing to perform the exercise program and wear the activity tracker
**Exclusion Criteria**	A potential subject who meets any of the following criteria is not eligible for enrolment into this study:<br>• Unstable bone metastases inducing skeletal fragility as determined by the treating clinician<br>• Untreated symptomatic known brain metastasis<br>• Estimated life expectancy < 6 months as determined by the treating clinician<br>• Serious active infection<br>• Too physically active (i.e. >210 minutes/week of moderate-to-vigorous intentional exercise) or engaging in intense exercise training comparable to the EFFECT exercise program<br>• Severe neurologic or cardiac impairment according ACSM criteria<br>• Uncontrolled severe respiratory insufficiency as determined by the treating clinician or if the patient is dependent on oxygen suppletion in rest or during exercise<br>• Uncontrolled severe pain<br>• Any other contraindications for exercise as determined by the treating physician<br>• Any circumstances that would impede adherence to study requirements or ability to give informed consent, as determined by the treating clinician<br>• Pregnancy
**Sample size considerations**	In the context of this study a positive result at the 6-month assessment of any of the two primary endpoints (physical fatigue, HRQoL) is of interest. To adjust for multiple testing, the Bonferroni-Holm method is considered. With n=139 patients per group (n=278 in total), for each endpoint separately a mean standardized effect size (ES) of at least 0.35 can be detected with an analysis of covariance (ANCOVA), adjusted for the pre-intervention levels of the outcome with a power of at least 78% or 82% at a (nominal) two-sided significance level of

	2.5%, assuming a correlation between pre- and post-intervention levels of Rho=0.3 or Rho=0.4, respectively.<br>To account for a potential drop-out rate of about 20%, a total number of n=350 patients will be enrolled into the study (175 per group).
**Ethics**	The study will be conducted in full conformance with the principles of the "Declaration of Helsinki" (64th WMA General Assembly, Fortaleza, Brazil, October 2013) or with the laws and regulations of the country in which the research is conducted, whichever affords the greater protection to the participant.

TABLE OF CONTENTS

OVERVIEW **3**

SYNOPSIS **5**

LIST OF ABBREVIATIONS AND RELEVANT DEFINITIONS **12**

1. INTRODUCTION AND RATIONALE **14**

2. OBJECTIVES **15**

2.1. Primary Objective 15

2.2. Secondary Objective(s) 15

2.3. Exploratory objectives 16

3. STUDY DESIGN **16**

4. STUDY POPULATION **17**

4.1. Inclusion criteria 17

4.2. Exclusion criteria 17

5. SAMPLE SIZE CALCULATION **18**

6. TREATMENT OF SUBJECTS **19**

6.1. Supervised exercise program 19

6.2. Control group 21

7. METHODS **23**

7.1. Study parameters 23

7.1.1. Main study parameter/endpoint 23

7.1.2. Secondary study parameters/endpoints 23

7.1.3. Exploratory endpoints 23

7.1.4. Safety endpoints 24

7.1.5. Other study parameters 24

7.2. Random allocation to intervention 24

7.3. Study Procedures 24

7.3.1. Cancer-related fatigue 26

7.3.2. Quality of Life 26

7.3.3. Additional secondary patient reported outcomes (questionnaires) 27

7.3.4. Body composition (Bio impedance, Anthropometrics) 27

7.3.5. Resting heart rate and blood pressure 27

7.3.6. Physical fitness/performance 28

7.3.7. Physical Activity 29

7.3.8. Satisfaction with the exercise intervention 30

7.3.9. Blood markers 30
7.3.10. Cost-effectiveness 31
7.3.11. Add-on measurements, only assessed in some centers 31
7.3.11.1. DEXA 31
7.3.11.2. CPET 32
7.3.11.3. Isokinetic and isometric peak torque 32
7.3.11.4. Muscle thickness with ultrasound protocol 32
7.4. Withdrawal of individual subjects 33
7.5. Temporary halt for reasons of subject safety 33
7.6. Replacement of individual subjects after withdrawal 33
7.7. Follow-up of subjects withdrawn from the intervention 33
7.8. Premature termination of the study 33
8. SAFETY REPORTING 34
8.1. (Serious) adverse events 34
8.1.1. Adverse events (AEs) 34
8.1.2. Serious adverse events (SAEs) 34
8.1.3. Follow-up of adverse events 35
8.2. Data Safety Monitoring Board (DSMB) 35
9. STATISTICAL ANALYSIS 35
9.1. Primary study parameter(s) 35
9.2. Secondary study parameters 36
9.3. Exploratory analysis 36
9.4. Cost-effectiveness analysis 36
9.5. Other study parameters 36
9.6. Interim analysis 37
10. ETHICAL CONSIDERATIONS 37
10.1. Regulation statement 37
10.2. Regulatory Authority/ Independent Ethics Committees 37
10.3. Recruitment and consent 37
10.4. Benefits and risks assessment, group relatedness 38
10.5. Compensation for injury 39
10.6. Incentives 39
11. DATA COLLECTION, HANDLING AND RECORD RETENTION 39
11.1. Recording of Data 39
11.2. Data Quality Assurance 40

11.3. Data Management 40
12. DEVIATION & VIOLATION 40
12.1. Deviation 41
12.2. Violation 41
12.3. Follow-up of Deviations and Violations 41
13. ADMINISTRATIVE ASPECTS, MONITORING AND PUBLICATION 41
13.1. Handling and storage of data and documents 41
13.2. Monitoring and Quality Assurance 42
13.3. Amendments 42
13.4. Annual progress report 42
13.5. Temporary halt and (prematurely) end of study report 42
13.6. Public disclosure and publication policy 42
14. REFERENCES 44
APPENDIX 1. Detailed description of the EFFECT exercise program 47
APPENDIX 2: Example of the training documentation of the supervised exercise Session 53

LIST OF ABBREVIATIONS AND RELEVANT DEFINITIONS

AE	Adverse Event
ANCOVA	Analysis of covariance
ACSM	American College of Sports Medicine
BIA	Bio Impedance Analysis
BPI	Brief Pain Inventory
CCMO	Central Committee on Research Involving Human Subjects (Centrale Commissie Mensgebonden Onderzoek)
CD	Cluster of Differentiation
CHMP	Committee for Medicinal Products for Human Use
CPET	Cardiopulmonary exercise testing
CTCAE	Common Toxicity Criteria for Adverse Events
CTLA	Cytotoxic T-lymphocyte-associated protein
DEXA	Dual-energy X-ray absorptiometry
DSMB	Data Safety Monitoring Board
ECG	Electrocardiogram
ECOG	Eastern Cooperative Oncology Group scale
eCRF	Electronic Case Report Form
EDGE	Evaluation Database to Guide Effectiveness
EMA	European Medicines Agency
EORTC	European Organisation for Research and Treatment of Cancer
ES	Effect Size
ESSI	The ENRICHD Social Support Instrument
EU	European Union
FAB scale	Fullerton Advanced Balance scale
FFM	Fat-Free Mass
FoxP3	Forkhead box P3
GDPR	General Data Protection Regulation
HIIE	High Intensity Intermittent Exercise
HR	Heart Rate
ICC	Intraclass Correlation Coefficient
ICER	Incremental Cost-Effectiveness Ratio
ICF	Informed Consent Form
ICIQ-UI	International Consultation on Incontinence Questionnaire Urinary Incontinence
IFNg	Interferon gamma
iMCQ	iMTA Medical Consumption Questionnaire
iMTA	Institute of Medical Technology Assessment
IL	Interleukin
iPCQ	iMTA Productivity Cost Questionnaire
MICT	Moderate-Intensity Continuous Training
MMRM	Mixed Models for Repeated Measures

**MNAR**	**Missing Not At Random**
**MSEC**	**Maximum Short Exercise Capacity**
**NWB**	**Non-Weight Bearing**
**PBMC**	**Peripheral Blood Mononuclear Cells**
**PCS**	**Pain Catastrophizing Scale**
**PHQ-4**	**Patient Health Questionnaire-4**
**PSQI**	**Pittsburgh Sleep Quality Index**
**QALYs**	**Quality Adjusted Life Years**
**QoL**	**Quality of Life**
**QWLQ_CS**	**Quality of Working Life Questionnaire for Cancer Survivors**
**RM**	**Repetition Maximum**
**RPE**	**Rated Perceived Exertion**
**RPM**	**Revolutions per Minute**
**(S)AE**	**(Serious) Adverse Event**
**SD**	**Standard Deviation**
**SOP**	**Standard Operating Procedure**
**Sponsor**	**The sponsor is the party that commissions the organisation or performance of the research. A party that provides funding for a study but does not commission it is not regarded as the sponsor, but referred to as a subsidising party.**
**TNFa**	**Tumor Necrosis Factor alpha**
**WB**	**Weight Bearing**
**W_{peak}**	**Peak Wattage**

1. INTRODUCTION AND RATIONALE

Metastases are the leading cause of breast cancer-associated deaths, responsible for more than 600.000 premature deaths around the world each year. One in eight women will develop breast cancer and up to 40% of the women diagnosed with early breast cancer will develop metastatic breast cancer [1]. Important drivers that determine how patients with metastatic breast cancer experience their HRQoL include levels of fatigue, pain, insomnia, depression and nausea. Of these side effects, fatigue (experienced by nearly up to 90% of individuals receiving cancer therapy) [2;3] exerts a deleterious impact on HRQoL and has a large negative impact on daily activities. As the overall percentage of women that survive 5 years following stage IV diagnosis, now reaching 30% with 10% surviving 10 years, is gradually increasing due to the fast development of new agents, larger number of women will potentially be confronted with diminished HRQoL and its sequelae.

Fatigue is a multidimensional side effect that manifests in different ways, i.e. as a physical, emotional and/or cognitive tiredness or exhaustion that limits the physical, functional, psychological and social well-being of patients. For this reason, fatigue is frequently mentioned as a pervasive and lingering side effect that tends to worsen during the course of treatment. As fatigue is such a major driver for the suboptimal HRQoL in patients with metastatic breast cancer and other advanced cancers, interventions that can successfully alleviate fatigue during their treatment are highly needed.

A systematic review of pharmaceutical and non-pharmaceutical interventions showed that exercise is the most promising intervention for reducing fatigue in cancer patients treated with curative intent [4]. However, to date no clear evidence has been delivered in patients with metastatic breast cancer. Only very few, mainly small studies with short interventions have been performed.

As exercise interventions have been predominantly studied in earlier stages of breast cancer, researchers within this project have identified the need for this type of research in patients with metastatic breast cancer. The condition of patient with advanced (metastatic) disease differs considerably from that of early-stage disease in terms of HRQoL and functional capacity.

There is ample evidence supporting the positive impact of therapeutic exercise on adverse effects of adjuvant treatment [5-8]. In this curative setting, exercise has shown positive results in patients undergoing treatment in terms of reducing fatigue, but also in improving psychological symptoms, such as depression and low self-esteem. Supervised moderate-to high intensity aerobic and/or resistance exercise programs consistently lead to lower fatigue as compared to usual care. In addition, beneficial effects on fitness, pain, sleep problems, nausea, self-esteem and also return to work have been reported. Interestingly, two studies investigated treatment tolerance and found that chemotherapy completion rates were higher in the exercise intervention group [9;10]. Also, adherence to these moderate-to high intensity exercise interventions was high, i.e. above 70% and the majority above 80%. Given the lack of alternatives for improving fatigue and the pressing unmet need to

improve HRQoL of advanced cancer patients, exercise represents a promising and desirable intervention.

Nevertheless, to convince stakeholders, such as health care professionals, patients and the health care insurers to adopt this non-pharmacological intervention, conclusive (cost) effectiveness evidence in the metastatic breast cancer setting is needed.

We hypothesize that a structured and individualised exercise program will diminish levels of physical fatigue and/or the overall HRQoL-related symptom burden in patients with metastatic breast cancer, and will be cost-effective from a societal perspective.

2. OBJECTIVES

2.1. Primary Objective

The primary aim is to assess the effects of a structured and individualised exercise intervention in patients with metastatic breast cancer (stage IV) on cancer-related physical fatigue and HRQoL. The primary endpoints will be assessed at six months. In addition, sustainability of effects (i.e. on primary endpoints) will be assessed at nine months.

2.2. Secondary Objective(s)

The secondary aim of the study is to assess the effects of a structured and individualised exercise intervention in patients with metastatic breast cancer (stage IV) on

- Separate HRQoL domains
- Breast cancer specific HRQoL symptoms
- Emotional, cognitive, and total fatigue scores
- Anxiety, depression
- Sleep
- Pain
- Cancer treatment-related toxicities grade≥3
- Physical fitness/performance (Steep ramp test; endurance test; 5-times sit to stand; short FAB scale; handgrip- and leg strength test; and in some centers also Cardiopulmonary exercise testing (CPET))
- Body composition (Bio impedance, anthropometrics, and in some centers also DEXA)
- Physical activity levels (questionnaire and activity tracker)
- Profiling of circulating white blood cell population and biomarkers (e.g., systemic inflammation, growth factors, blood/brain barrier modulators)
- Quality Adjusted Life Years (QALYs), healthcare costs, productivity costs, patient- and family costs. These data will be used to estimate cost-effectiveness of the intervention.

- Quality of working life (add-on in several centers)
- (Serious) Adverse Events potentially related to exercise intervention

2.3. Exploratory objectives

- Overall and breast-cancer specific survival
- Progression-free survival
- Body composition (maximal isokinetic and isometric leg strength, muscle thickness of m. rectus femoris (RF) and m. vastus lateralis (VL) (add-on in one center)
- Urinary incontinence

3. STUDY DESIGN

The EFFECT study is a multinational, randomised controlled trial with two study arms designed to assess the effects of a structured and individualised 9-month exercise intervention in patients with metastatic breast cancer (stage IV) on HRQoL, fatigue and other disease and treatment-related side effects. The control group will receive usual care, an activity tracker and a brief physical activity advice.

The study setting will be clinical centres, local fitness centres and physical therapy practices.

The primary endpoints will be assessed at six months. In addition, at nine months sustainability of effects and cost-effectiveness will be assessed. Patients will be followed for disease progression and survival for five years beyond the 9-month study period.

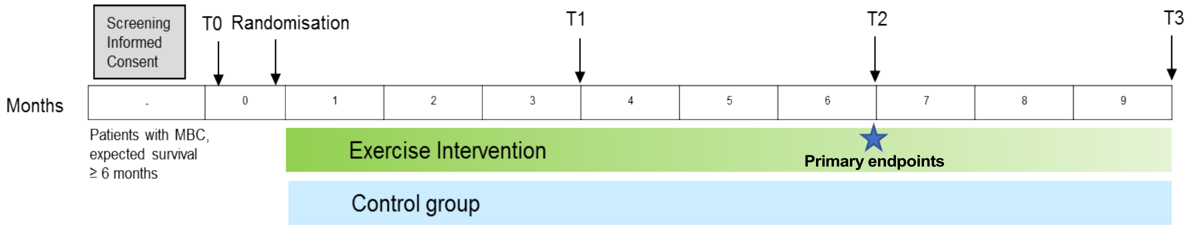

In total, 350 patients will be recruited. We aim to recruit 310 patients from approximately seven centres from the following European countries: Germany *(2 centres)*, the Netherlands (*2 centres*), Poland, Spain, and Sweden. In addition, 40 patients in Melbourne, Australia, will be recruited.

Inclusion and all measurements will be performed in these eight centres. If needed, we will extend the number of centres.

The exercise program will be executed by local physiotherapists, exercise physiologists or fitness instructors, who will be trained to deliver the intervention as intended, near the patient's home.

We expect to include on average about two participants per centre per month, approximately 16 patients per month in total (across all eight sites). As a result, we expect to include the 350 participants over a period of two years.

4. STUDY POPULATION

Three hundred and fifty patients with metastatic breast cancer will be recruited from eight clinical centres in Germany, the Netherlands, Poland, Spain, Sweden and Australia.

The EFFECT study will recruit both female and male patients; male breast cancer patients are expected to make up a very small minority, namely 1% of the total number of participants.

4.1. Inclusion criteria

In order to be eligible to participate in this study, a subject must meet all of the following criteria:

- Age ≥ 18 years
- Diagnosis of breast cancer stage IV
- ECOG (Eastern Cooperative Oncology Group scale) performance status ≤ 2
- Able and willing to perform the exercise program and wear the activity tracker

4.2. Exclusion criteria

A potential subject who meets any of the following criteria will be excluded from participation in this study:

- Unstable bone metastases inducing skeletal fragility as determined by the treating clinician
- Untreated symptomatic known brain metastasis
- Estimated life expectancy < 6 months as determined by the treating clinician
- Serious active infection
- Too physically active (i.e. >210 minutes/week of moderate-to-vigorous intentional exercise) or engaging in intense exercise training comparable to the EFFECT exercise program
- Severe neurologic or cardiac impairment according ACSM criteria
- Uncontrolled severe respiratory insufficiency as determined by the treating clinician or if the patient is dependent on oxygen suppletion in rest or during exercise
- Uncontrolled severe pain
- Any other contraindications for exercise as determined by the treating physician
- Any circumstances that would impede adherence to study requirements or ability to give informed consent, as determined by the treating clinician
- Pregnancy

5. SAMPLE SIZE CALCULATION

In the context of this study a positive result at the 6-month assessment of any of the two primary endpoints (physical fatigue, HRQoL) is of interest. To adjust for multiple testing, the Bonferroni-Holm method is considered. With n=139 patients per group (n=278 in total), for each endpoint separately a mean standardized effect size (ES) of at least 0.35 can be detected with an analysis of covariance (ANCOVA) adjusted for the pre-intervention levels of the outcome with a power of at least 78% or 82% at a (nominal) two-sided significance level of 2.5%, assuming a correlation between pre- and post-intervention levels of Rho=0.3 or Rho=0.4, respectively.

However, the probability for at least one of the two tests corresponding to the two primary endpoints to yield a significant result, if both alternative hypotheses are true, is much higher than the values given in Table 1 (unless the tests are perfectly correlated). Taking also repeated measures into account using mixed models might further increase the power. The anticipated effect size of 0.35 is based on results from six randomized exercise intervention trials with breast cancer patients in the adjuvant treatment setting (including four studies performed by consortium members). Pooled analysis of that data (n=784) showed significant beneficial exercise effects on physical fatigue (ES: 0.35, 95% CI 0.21, 0.49) [6].

The sample size of n=278 also allows exploratory moderator or subgroup analyses that will give further insights into which patient populations might benefit most from exercise.
To account for a potential drop-out rate of about 20%, a total number of n=350 patients will be enrolled into the study(175 per group). As we exclude patients with an estimated life expectancy < 6 months, the number of deaths before the primary assessment is expected to be low, and since the questionnaire-based primary endpoints can be completed with relatively little effort, the overall drop-out rate is expected to be below 20%.

The small male sample size will presumably not allow for subgroup analyses in men; however, outcomes will be generalised to both genders.

Table 1: **Sample size estimations (per group) based on correlations measures, effect size and power**

	Rho=0.3			Rho=0.4		
	Effect size			Effect size		
Power	.3	.35	.4	.3	.35	.4
60	128	94	73	118	87	67
62	133	98	76	123	91	70
64	139	102	79	128	95	73
66	144	107	82	133	98	76
68	150	111	85	139	102	79
70	157	116	89	145	107	82
72	163	120	93	151	111	86
74	170	126	97	157	116	89
76	178	**131**	101	164	121	93
78	186	137	105	171	126	97
80	194	143	110	179	**132**	102
82	203	150	115	188	139	106
84	214	157	121	197	145	112

Rho= correlation between pre- and post-intervention measures

6. TREATMENT OF SUBJECTS

In the EFFECT study, proven effective exercise interventions from the curative setting are adapted to the metastasized setting. The intervention exists of:

0-6 months:	Supervised exercise program: 60 min twice a week<br>Unsupervised exercise program: 30 min per day on all remaining days of the week (5 days per week) supported by an activity tracker (Fitbit) and an exercise App
6-9 months:	Supervised exercise program: 60 min once a week<br>Unsupervised exercise program: 60 min once a week and 30 min per day on all remaining days of the week (5 days per week) supported by an activity tracker (Fitbit) and an exercise App

6.1. Supervised exercise program

Prior to commencing the program, each participant will have an intake session in order to individualise the standard exercise prescription to their specific needs. If patients are at risk for cardiovascular disease according to ACSM screening criteria, medical clearance will be sought by means of an exercise electrocardiogram (ECG) performed by a sports physician or cardiologist, unless

a recent exercise ECG is available (within three months from baseline). The exercise program will start at least one week after the baseline visit to be able to collect pre-intervention physical activity data (activity tracker).

The exercise program will start with a 6-month period, where patients will participate in a supervised multimodal exercise program twice a week (60 minutes per session) and additional unsupervised exercises. After completing the initial 6-month period, one supervised session will be replaced by one unsupervised session until month nine.

The multimodal exercise program comprises aerobic-, resistance- and balance components. The supervised exercise sessions will be offered at various community or hospital-based fitness centres, physical therapy practices, gyms or personal training facilities throughout the catchment areas of the recruiting sites and close to the patients' homes. The supervised sessions will be conducted by fitness instructors, exercise physiologists or (oncology) physiotherapists who are trained by the study team to ensure a sound and safe execution of the program, according to Appendix 1. The same procedure has already been successfully used in other exercise studies of our consortium in cancer patients in the curative setting [10-14].

The aerobic exercise component includes moderate to vigorous intensity cardiovascular exercise. Participants will progress from moderate intensity continuous exercise to high intensity intermittent exercise (HIIE; intervals of high and low intensity exercise), if possible/ desirable. The resistance exercise component involves exercises that target the major upper and lower body muscle groups. Intensity will be extrapolated from 12 repetition maximum (RM) tests (i.e. the maximal weight that can be lifted 12 times which is equivalent to ~70% of 1RM) using 1-3 sets per exercise. In case of bone metastases, specific exercises might need modification. Next to the standard resistance training protocol, a table for selecting appropriate and safe exercises will be provided to all exercise supervisors. In addition, supervisors can directly contact the EFFECT exercise expert board in case of questions. This expert board consists of oncologists, physiotherapists and exercise experts from all study centres.

The balance exercise component includes exercises that involve a variety of different stances and will be progressed by including more challenging tasks. Aerobic, resistance and balance exercise prescription will be progressive and modified according to individual response (Appendix 1).

If the training facilities are closed or patients do not want to visit them because of the Corona situation, an alternative live online training program will be provided. The live online training sessions will be instructed by a qualified exercise trainer and include the same components as the face-to-face training program. The exercise sessions will take place two times per week with a maxi-mum of nine participants per session in order to guarantee adequate patient care. The study team will provide the patients with the required training equipment free of costs (e.g. resistance bands and free weights for resistane training; and a hometrainer (preferable) or an aerobic step for endurance training). Prior to the start of the live online training program, the exercise trainer will

conduct one individually guided face-to-face training session in which the exercises, training equipment and training documentation are explained. To ensure a safe execution of the program, the exercise trainer will ask about the occurence of adverse events before the start of each live online training session in a private level and will closely monitor each participant during the session to ensure that all exercises are performed correctly. For the event of an injury or emergency, the trainer has an address list of the patients' homes/training locations and a telephone in close proximity to be able to make an emergency call.

Unsupervised exercise program

In addition to the supervised exercise program, patients will be encouraged to exercise for at least 30 minutes per day on all remaining days of the week. These exercises will be supported by an activity tracker (Fitbit) and a corresponding exercise App, which is specifically designed for the EFFECT trial. The supervisors will be clearly informed about the unsupervised exercises so that they can support the patient being active in their daily life.
Additionally, one supervised session will be replaced by an unsupervised session of 60 minutes in the last three months of the program. Patients will be guided by the App for this training session. The app-based training program partly includes exercises that patients will have learnt during the supervised program. The exercises are illustrated with simple animations. To increase motivation for the unsupervised training sessions, the App includes innovative elements like avatar-based training and gaming aspects. The patients will be rewarded with points if exercises are performed on a regular basis. These points improve the wellbeing and joyfylness of a pet (gaming aspect) based on the Tamagotchi concept.
The patients in the intervention group will be instructed by the study team about the use of the App and will also receive written information. Additionally, the exercise supervisors will be trained to assist the patient with use of the App. If patients do not have an appropriate mobile phone, the study will provide one for study use only.

Attendance and compliance to the exercise program
During the intervention period, the supervisor will register patient’s attendance. After one week no show, the supervisor or the study team will call the patient.
Patients register their compliance with all parts of the exercise intervention in weekly training logs (Appendix 2). In addition, physical activity data of the activity tracker and the App will provide insight into the compliance to both the supervised and the unsupervised exercise training sessions. After 3-4 months of the intervention, a researcher will perform a monitor visit either via Skype or in person with the supervisor and the patient, dependent on the specific patients’ situation.

6.2. Control group

Patients randomized to the control group will receive standard medical care; exercise is not part of this yet. Additionally, they receive an activity tracker (like the intervention group) but without spe-

cific instructions. We will advice control patients to avoid inactivity and be as physically active as current abilities and conditions allow, with the aim to progress towards being physically active for 150min/week in line with the current physical activity guidelines.
We decided to provide the activity tracker and give the advice, since it has been shown that providing something to the control group decreases the risk of drop-out and of contamination (i.e. adoption of the intervention by controls)[15]. Patients who consent to participate in an exercise intervention trial are generally willing to exercise, and activity trackers have been observed to provide a low-level stimulus to engage in physical activity. Thus, the EFFECT trial will assess whether the exercise intervention is significantly better than a simple low-level physical activity stimulus. All control group patients will follow the same study visit schedule.

7. METHODS

7.1. Study parameters

7.1.1. Main study parameter/endpoint

Primary endpoints of the EFFECT study are
1) Cancer-related physical fatigue (EORTC QLQ-FA12)
2) Health-related Quality of Life (EORTC QLQ-C30 Summary Score)

7.1.2. Secondary study parameters/endpoints

- Separate HRQoL domains (EORTC QLQ-C30 function and symptom scores)
- Breast cancer specific symptoms (EORTC QLQ-BR45)
- Emotional, cognitive, and total fatigue scores (EORTC QLQ-FA12)
- Anxiety and depression (PHQ-4)
- Pain (BPI, painDETECT, PCS)
- Sleep (PSQI)
- Cancer treatment-related toxicities grade≥3 (Common Toxicity Criteria for adverse events (CTCAE))
- Physical fitness/performance (Steep ramp test; endurance cycle test, 5-times sit to stand, short FAB scale, handgrip- and leg strength test; and in some centers also Cardiopulmonary exercise testing (CPET))
- Body composition (Bio impedance, anthropometrics, and in some centers also DEXA)
- Physical activity levels (Physical activity questionnaire; activity tracker (Fitbit))
- Profiling of circulating white blood cell populations and biomarkers (e.g., systemic inflammation, growth factors, blood/brain barrier modulators)
- Cost-effectiveness parameters: (Quality Adjusted Life Years (QALYs) (EQ-5D-5L), iMCQ (healthcare costs) and iPCQ (productivity costs))
- Quality of working life (add-on in several centers)

7.1.3. Exploratory endpoints

- Overall and breast-cancer specific survival: time from study inclusion to (all-cause or breast cancer specific) death – medical records and/or cancer registry.
- Progression-free survival: time from study inclusion to disease progression or death from any cause- medical records and/or cancer registry
- Body composition measured with, maximal isokinetic and isometric leg strength, muscle thickness of m. rectus femoris (RF) and m. vastus lateralis (VL) (add-on in one center)
- Urinary incontinence (add-on in several centers)

7.1.4. Safety endpoints

- (Serious) Adverse events potentially related to the exercise intervention

7.1.5. Other study parameters

- Socio-demographics
- Social support (ESSI)
- Medical history and concomitant diseases
- Concomitant medication and diseases
- Breast cancer characteristics and treatment history
- Satisfaction with the exercise intervention (intervention group only)

7.2. Random allocation to intervention

Patients will be allocated by central computerized randomization (using a blocked computer-generated sequence) in a 1:1 ratio to either the intervention or control group. Randomisation will be stratified by centre and therapy line (first/second versus higher). The randomisation procedure will be conducted by a study member in CASTOR® (eCRF) after Informed Consent and baseline measurements.

Due to the nature of the intervention, it is not possible to blind the patients, the local study nurses, or the investigators to the treatment assignment. The biomarker analyses are performed blinded since laboratory personnel are not informed of treatment assignment.

7.3. Study Procedures

Patients will visit the clinical centre for measurements at baseline, at three months and six months after baseline. The primary outcome assessment is at six months. For patients undergoing chemo-therapy, measurements will take place at least three days after chemotherapy infusion.

Patients will be asked not to exercise vigorously or drink alcohol 24 hours before each visit.

The primary measurements at these visits comprise cancer-related fatigue and HRQoL assessed by questionnaires. Additionally, body composition measures, heart rate, blood pressure, physical fitness testing, various questionnaires and blood sampling will be performed. Not all measurements have to be performed on one day, but within one week. Order of the measurements will be standardized in SOPs, e.g. blood sampling and body composition measurements will always take place before exercise testing. See Table 2 for an overview of all the measurements.

To assess sustainability of effects at nine months, patients will be sent a set of online questionnaires at nine months after baseline and the study team will call the patients to ask for exercise-related adverse events and changes in concomitant medication.

Table 2: overview of the measurements in the EFFECT trial

	T0	T1	T2	T3
	Baseline	Month 3<br>± 14 days	Month 6<br>± 14 days	Month 9<br>± 21 days
Socio-demographics	X			
Social Support (ESSI)	X			
Medical history and concomitant diseases (log-form)	X	X	X	X
Concomitant medication (Log-form)	X	X	X	X
Cancer-related Fatigue (EORTCQLQ-FA12)	X	X	X	X
Health-Related Quality of life (EORTC QLQ-C30/-BR45)	X	X	X	X
Depression and anxiety (PHQ-4)	X	X	X	X
Sleep (PSQI)	X	X	X	X
Pain (BPI, painDETECT, PCS)	X	X	X	X
Physical activity questionnaire + activity tracker	X	X	X	X
Blood sampling	X	X	X	
Height	X			
Anthropometrics, Bio impedance, and in some centers also DEXA	X	X	X	
Resting heart rate/ blood pressure	X	X	X	
Excercise program (weekly for 9 months)*	X	X	X	X
Physical fitness/ performance measurements (Steep ramp test, endurance cycle test, 5-times sit to stand, short FAB scale, handgrip- and leg strength test; and in some centers also Cardiopulmonary exercise testing (CPET))	X	X	X	

Work status/ healthcare resources consumption (iPCQ/iMCQ)		X	X	X
Quality of working life (QWLQ-CS) (in some centers)	X	X	X	X
Urinary incontinence (ICIQ-UI) (in some centers)	X	X	X	X
Assessment of intervention costs	X	X	X	
EQ-5D-5L	X	X	X	X
Exercise-related AEs		X	X	X
Satisfaction with exercise intervention*		X	X	X
Isokinetic and isometric legstrength (peak torque) / Muscle thickness with ultrasound protocol (measured in one center)	X	X	X	
Cancer characteristics and treatment history	extract from medical documentation (at T0)			
Cancer progress and treatment over the course of the study	extract from medical documentation			
Cancer treatment related toxicities (CTCAE)	extract from medical documentation			

*Only for the exercise group

7.3.1. Cancer-related fatigue

Cancer-related physical fatigue will be measured at all visits using the questionnaire of the European Organisation for Research and Treatment of Cancer (EORTC) that has been specifically developed and validated for cancer-related fatigue (EORTC QLQ-FA12) [16]. Scores for physical fatigue will be calculated according to the EORTC manual.

7.3.2. Quality of Life

Health-related Quality of Life will be calculated using the new EORTC QLQ-C30 Summary Score (at all study visits) recommended by the EORTC Quality of Life Group, which has been recently developed and evaluated [17]. The Summary Score is calculated as follows:

QLQ-C30 Summary Score = (Physical Functioning+ Role Functioning+ Social Functioning+ Emotional Functioning+ Cognitive Functioning+ 100-Fatigue+ 100-Pain+ 100-Nausea_Vomiting+ 100-Dyspnoea+ 100-Sleeping Disturbances+ 100-Appetite Loss+ 100-Constipation+ 100-Diarrhoea)/13.

The Summary Score should only be calculated if all of the required 13 scale scores are available (using scale scores based on the completed items, provided that at least 50% of the items in that scale have been completed. https://qol.eortc.org/app/uploads/sites/2/2018/02/scoring_of_the_qlq-c30_summary_score.pdf)

Metastatic breast cancer patients comprise a heterogeneous group which experiences a variety of other side effects besides fatigue, e.g. pain, nausea, dyspnoea, impaired physical, emotional, or social function that all together can severely impact their HRQoL. These aspects are all assessed by the EORTC QLQ-C30 and included in the Summary Score. Thus, a significant benefit of the exercise intervention on the EORTC QLQ-C30 Summary Score would be of significant clinical relevance for patients with metastatic breast cancer.

7.3.3. Additional secondary patient reported outcomes (questionnaires)

Key secondary outcome measures comprise the single EORTC QLQ-C30/-BR45 functions and symptoms, the other fatigue dimensions of the EORTC QLQ-FA12 (emotional, cognitive, and total fatigue scores), pain (BPI, painDETECT, PCS), sleep (PSQI), anxiety , depression (PHQ-4) and working life (Quality of Working Life Questionnaire for Cancer Survivors (QWLQ-CS; [26]). Regarding the EORTC physical functioning subscale, we added four items from the EORTC itembank ([27]) in order to assess a wider range of physical functioning. In addition, three questionnaires that are needed to perform cost-effectiveness analysis are provided, i.e. EQ-5D-5L (basis for estimation of Quality Adjusted Life Years), iMCQ (health care resources use and patient- and family costs) and iPCQ (work status and productivity losses due to disease or treatment). The above mentioned questionnaires that will be used are validated and show satisfying psychometric properties. Most questionnaires are available in the languages of the participating countries i.e. Dutch, English, German, Polish, Swedish and Spanish. For those parts of the assessment package not available in all languages, we employed the EORTC procedures for translating questionnaires, using a vigorous forward (from English) – backward (to the target language) process.
Patients will complete these questionnaires online on a computer or tablet at home or at the research centre depending on the usual procedures of the clinical centre. Additionally, there will be a paper-pencil option. Also for questionnaires not approved for ePRO use.

7.3.4. Body composition (Bio impedance, Anthropometrics)

Anthropometrics (height, weight, waist- and hip circumference) will be measured in light clothing without shoes using standardized procedures described in study SOPs. To assess body composition (fat mass and fat free mass), we will use whole body/ segmental bio impedance measurement (BIA) according to standardized procedures. Fat-Free Mass (FFM) will be calculated with the Kyle equation using Resistance and Reactance at 50kHz measured by the BIA [18]. Scales and BIAs may differ between centres, but patients within each centre will consistently be measured on the same device.

7.3.5. Resting heart rate and blood pressure

Patients' resting heart rate will be measured after a five minute rest in the supine position.

Blood pressure will be measured twice. The first measurement will be taken with the patient in the supine position. The second measurement will be taken with the patient sitting quietly in a chair with back supported, feet on the ground and arm supported at heart level. The patient should rest in the respective position for at least one minute prior to the measurement.
As the first measurement per position appears to be high to normal (systolic blood pressure 120-129mmHg and/or diastolic blood pressure 80-89mmHg) or high (systolic blood pressure >130mmHg and/or diastolic blood pressure >90mmHg), the measurement will be repeated after one minute rest and the mean value will be recorded.
Patients with severe hypertension at rest (systolic blood pressure >180mmHg and/or diastolic blood pressure >110mmHg) require further medical evaluation prior to proceeding with physical fitness testing.

7.3.6. Physical fitness/performance

The order of the physical fitness and performance measurements are standardized.

Maximal Short Exercise Capacity

Maximal Short Exercise Capacity will be measured with the *Steep Ramp Test* using a cycle ergometer. This is a maximal test but, due to its steep increments, the limiting factor is peripheral muscle strength, and not cardiovascular capacity. This makes the test safe to perform without ECG in many populations, including cancer patients [19].The test starts after 3 minutes of unloaded cycling, at 25W, and is increased by 2.5W per second. Patients are instructed to cycle at ~70 RPM. The test ends when cycling frequency drops below 60 RPM. The outcome is registered as the highest achieved output in Watts and is referred to as MSEC (maximum short exercise capacity). From the MSEC, peak Wattage (W_{peak}) can be estimated using a regression equation [20]. Additionally, we record RPE at termination, time cycled and heart rate 1 and 2 min after termination.

Aerobic capacity

Aerobic capacity will be assessed using an *endurance test* on a cycle ergometer with a fixed load. The fixed load will be determined as 70% of the estimated peak Wattage (W_{peak}) at baseline using the Steep Ramp Test (see above). For patients who stopped the Steep Ramp Test early and there are objective reasons to believe that a maximal MSEC was not achieved, 80% of the estimated W_{max} will be considered to avoid a ceiling effect of the test. However, if the Steep Ramp Test was stopped early due to pain or discomfort, 70% of the estimated W_{max} will be used for safety precautions.
Patients will be asked to maintain a speed of ~70 RPM. The test ends when RPM drops below 60. The following parameters will be registered: time cycled in minutes, Borg Score of perceived exertion at termination and heart rate 1 and 2 min after termination.

Physical performance

Physical performance will be assessed using the 5-times sit to stand test [21] and the short FAB scale [28,29]. The 5-times sit to stand test assesses functional strength of the lower limbs. The test score is the time (in seconds) needed to complete 5 complete chair stands. The short FAB scale is a group of measures that combines the results of both dynamic and static balance exercises. This scale is mainly intended to identify balance problems of varying severity in functionally independent older adults' who are at an increased risk to experience fall-related injuries due to sensory impairments. This scale has a good reliability (0.96-0.98) [30,31] and has been highly recommended by the EDGE task force on oncological physical therapy. The scores range from 0 (worst performance) to 16 (best performance).

Muscle strength

To get an indication of muscle strength of the patient, we will assess hand grip strength and leg strength. Upper extremity grip strength will be assessed using a handgrip dynamometer (hydraulic Jamar®). The participant will be asked to squeeze the dynamometer as hard as possible with each hand. The best of three attempts will be recorded. Lower extremity muscle strength (quadriceps or knee extensors) will be assessed using a leg-press hypothetitical 1-RM test according to a standardised protocol or an isokinetic evaluation and training machine (Isomed 2000®), unless there are bone metastases that prohibit safe testing. Maximal isokinetic peak torque (MIPT) will be tested for each leg at 60°/s using the Isomed 2000®.

7.3.7. Physical Activity

The level of physical activity will be measured subjectively by a questionnaire (all visits). In addition, physical activity will be monitored objectively by an activity tracker (Fitbit inspire HR) that participants are asked to wear during the study. The study personnel will motivate patients to wear the activity tracker as much as possible since data will be extracted from the tracker during the whole study period. However, since the periods for main comparison between groups are: seven days after randomisation (T0, before start of the intervention), and seven days before T1 (3 months), T2 (6 months) and T3 (9 months), the participants will be kindly asked to constantly wear the tracker during these time periods.

The physical activity questionnaire consists of the Modified Version of the Godin-Shephard Leisure-Time Exercise Questionnaire [22, 23] and complementary questions, on types and settings of exercising, in order to get a complete picture of the patients' physical activity level. The Godin- Questionnaire is a short questionnaire that is often used to assess leisure time physical activity in oncology research [24]. It is a 4-item self-administered questionnaire, including questions on the average frequency and duration one engages in mild, moderate and vigorous aerobic activities and moderate to intense resistance exercises in bouts of at least 10 min duration during leisure time in a typical week.

7.3.8. Satisfaction with the exercise intervention

At three, six and nine months, we will assess the satisfaction with the exercise intervention by means of a self-designed questionnaire. The questionnaire contains satisfaction items with regard to the supervised program, the trainer, the activity tracker and the supporting app.

7.3.9. Blood markers

Blood sampling will be performed by vein puncture and will take place at baseline, after three and six months. Plasma, serum, buffy coat and peripheral blood mononuclear cells (PBMC) will be collected at rest (30 mL). All tubes for all centres will be provided by Karolinska Institute, Sweden. In the 24 hours prior to blood sampling, patients are instructed not to exercise vigorously or drink alcohol, and in the 2 hours prior to blood sampling, they are asked to abstain from cigarettes, food and drinks. Participants will not be tested for special conditions, such as pregnancy or HIV.

Immediately after collection, the blood samples will be centrifuged and stored at -80°C at the local laboratory according to standardized procedures. Blood samples will be transported to the central biobank at the Karolinska Institute after the last sample has been taken locally for the analysis specified below.

Analyses

Blood will be sampled to investigate the effects of the exercise intervention on blood markers and to elucidate: 1) mechanisms by which physical exercise may affect outcomes, 2) markers associated with prognosis and 3) pathophysiology of fatigue and other HRQoL-related outcomes
in patients with metastatic breast cancer. We will profile circulating white blood cell populations and biomarkers (e.g., systemic inflammation, growth factors, blood/brain barrier modulators).

In order to identify novel biomarkers responsive to the EFFECT intervention in this patient population, collected serum samples will be run on the Olink PEA platform (Oncology and Inflammation Array) that provides information on 92 oncology related cytokines and growth factors. In addition, selected factors of specific interest (such as IFNg, IL-1, IL-2, IL-4, TNFa) will be run on the Merck-Millipore custom HCYTOMAG platform for increased sensitivity and validation. Both platforms as well as technical expertise are readily available at the Science for Life Laboratory in Stockholm, Sweden.

In order to describe the population distribution and functional properties of the circulating leukocyte populations, we will apply flow cytometry analysis on the live frozen PBMCs. Relating the number of cells with surface markers such as CD4, NK1.1, CD8 to the total CD45+ population, we will provide

information on the population distribution. Flow cytometry analyses of markers such as FoxP3, CTLA and Granzyme B offers information on functionality.

7.3.10. Cost-effectiveness

The actual costs incurred with both strategies (intervention versus control) will be compared up until nine months after randomisation. Cost estimates will be based on the actual costs in both study arms. The cost analysis will be done from a societal perspective, implying that all costs are included, irrespective of the party that bears the costs. As the societal perspective includes healthcare costs, this extensive cost analysis also facilitates an analysis from the healthcare perspective, for those countries that prefer the healthcare perspective over the societal perspective. Three major types of costs are included in the societal perspective, i.e. healthcare costs, patient and family costs and productivity costs. Direct costs include costs of the exercise program, e.g. the intake cost, activity trackers, use of the app and wage costs of a supervisor. Other direct costs include costs of health service utilization such as general practitioner and oncologist contacts, cancer treatment, hospitalisation and rehabilitation. Patient and family costs include own expenses and travel costs, as well as time costs of family members that are needed to help the metastatic breast cancer patient, for instance driving the patient to exercise therapy. Furthermore, work status and absence from work due to illness and its treatment will be administrated.

Patients will be asked to complete questionnaires on these different types of costs, with the aim to collect data on the units of resources used by these patients, e.g. how often patients consulted general practitioners and medical specialists within the hospital setting. These questionnaires will be completed by patients three, six and nine months. We will use standardized questionnaires, the iMCQ (healthcare-, patient- and family costs) and iPCQ (productivity costs). Costs will be calculated by multiplying units of resource use with standard reference prices for those units (if available within a participating country). Should no standard reference prices be available it will be necessary to perform own costing research. For the valuation of productivity losses, the friction costs method will be used in baseline analyses, with the concurring Human Capital Method, that may be the preferred method in some participating countries, to be used in sensitivity analyses.

7.3.11. Add-on measurements, only assessed in some centers

7.3.11.1. DEXA

Regional and whole body lean mass and fat mass will be derived from whole body dual-energy X-ray absorptiometry (DEXA) scans. Trunk adiposity, visceral fat and adipose indices will be assessed using standard procedures.
DEXA scans are routine clinical tests but carry a small risk to the patient. DEXA involves exposure to radiation. The level of radiation exposure is exceedingly small (10-30 microSieverts uSv]) in comparison to the natural annual radiation dose in western communities (approximately 3000 uSv). A person would receive radiation exposure of approximately 80 uSv on an airline flight of 8 hours or 30 to 40 uSv during a typical x-ray.

7.3.11.2. CPET

An add-on measurement will be Cardiopulmonary exercise testing (CPET), in order to assess the cardiorespiratory fitness of the participating breast cancer patients.
The CPET procedure will start at 20 watts with work rate increasing by 10 W per min until voluntary exhaustion. The participants will be encouraged to pedal at a cadence between 60 and 80 rpm and to spend maximal effort. Heart rate and gas exchange will be recorded continuously. Peak heart rate and peak oxygen consumption will be considered the highest 20 sec average values during or immediately post exercise. Maximal respiratory exchange ratio will be considered the highest 20 sec average values during exercise. Blood samples for the determination of peak blood lactate concentration will be taken before, at exercise termination and after 3 min of recovery. Blood pressure will be monitored every two minutes. Peak power output will be protocoled as well. RPE will be recorded every 2 min and at exercise termination.

7.3.11.3. Isokinetic and isometric peak torque

Maximal isokinetic and isometric strength is assessed unilaterally, in both legs, using an isokinetic dynamometer. The tests are performed in the right leg first, and then in the left leg (OBS! The order can be randomized instead). Measures of concentric peak torque are obtained at constant velocities of 60 and 180°/s. Subjects perform two sets of two maximal actions at each velocity (30 s rest between sets at the same speed; 2 min recovery between different speeds), and the best result represents peak torque. After 2 minutes of rest, maximal isometric torque is measured at a knee angle of 120°. Subjects are instructed to push with maximal effort for ~5 s. Two trials with 60 s rest between attempts are allowed. The best score in a 1-s window defines peak isometric torque.

7.3.11.4. Muscle thickness with ultrasound protocol

Thickness of both m. rectus femoris (RF) and m. vastus lateralis (VL) is measured using ultrasonography. The ultrasound machine is operated using its default gain levels within the “skeletal muscle” mode. The subject is resting in supine position on an examination bed with the knee in full extension (i.e. anatomical zero). The right thigh is assessed first, followed by the left side. The scanning site is located by bisecting the distance between the anterior superior iliac spine and the superior pole of the patella. The transducer is placed transversally to the thigh. RF thickness is measured first, followed by VL, as the distance between superficial and deep aponeuroses. Two different pictures are taken. If the values differ >3%, a third image is obtained. As a precaution to avoid any pressure by the probe on the muscle that could bias the measurement, ample amounts of water-soluble transmission gel are applied. Pictures are considered valid when the normal curved shape on the superficial part of the muscle is clearly visible.
Echogenicity (expressed as grayscale values; from 0 to ~255 in arbitrary units) of RF and VL is assessed in saved pictures using imageJ or another image-processing software. After selecting the region of interest within the superior and inferior fascial borders of the muscle, the grayscale is assessed in the same number of images used for muscle thickness.

7.3.11.5. Urinary incontinence

The ICIQ-UI Short Form[32] is a brief (four items) and psychometrically robust patient-completed questionnaire on urinary incontinence. The first three items (frequency, amount and impact on everyday life) sum up to a score ranging from 0-21. The last item assesses in addition when does urine leak. Patients will be asked to complete the first item (frequency) at each visit. If the patient answers "never" no further questions will be asked. Otherwise the patient is asked the remaining three items of the ICIQ-UI.

7.4. Withdrawal of individual subjects

Participants can leave the study at any time, for any reason, if they wish to do so without any consequences. The investigator can decide to withdraw a participant from the intervention and/or the study for urgent medical reasons.

7.5. Temporary halt for reasons of subject safety

The sponsor will suspend the study if there is sufficient ground that continuation of the study will jeopardise subject health or safety. The sponsor will notify the accredited ethics committee without undue delay of a temporary halt including the reason for such an action. The study will be suspended pending a further positive decision by the ethics committee. The investigator will take care that all subjects are kept informed.

7.6. Replacement of individual subjects after withdrawal

Subjects that have withdrawn, after baseline data completion, will not be replaced.

7.7. Follow-up of subjects withdrawn from the intervention

Patients in the exercise intervention group that discontinue the exercise program will be asked to complete the assessments (at least the online questionnaires) as far as possible (at home and/or at the study centre).

7.8. Premature termination of the study

The study will be terminated prematurely when recruitment is far behind schedule and does not increase after multiple rescue measures.

8. SAFETY REPORTING

8.1. (Serious) adverse events

8.1.1. Adverse events (AEs)

Adverse events are defined as any undesirable experience occurring to a subject during the study, whether or not considered related to the study. Adverse events potentially related to the study (i.e. to the exercise intervention and study measurements) reported by the subject, observed by the trainer or study personnel will be recorded. We will record adverse events potentially related to physical activities/exercise or study exercise or study assessments and restrict these to events that:

- required treatment or talking to a doctor or other health professional
- caused any other persistent worsening of patients' health or well-being, new occurrence of substantial pain or swelling e.g. muscle tears
- occured during or after a supervised session and required restrictions/alterations or early termination of the training, or any treatment or clarification by a physician

Patients in both groups will be asked by the study team about exercise- and study measurement related AEs systematically and in a standardized manner at T1, T2, and T3. In addition, patients in the exercise intervention group will be asked by their trainer before and after each supervised session whether any potentially exercise-related AEs occurred during or since the last session.

8.1.2. Serious adverse events (SAEs)

A serious adverse event is any untoward medical occurrence or effect that:

- results in death;
- is life threatening (at the time of the event);
- requires hospitalisation or prolongation of existing inpatients' hospitalisation;
- results in persistent or significant disability or incapacity;
- Any other important medical event that may not result in death, be life threatening, or re-quire hospitalization, may be considered a serious adverse experience when, based upon ap-propriate medical judgement, the event may jeopardize the subject or may require an inter-vention to prevent one of the outcomes listed above.

The investigator will only report exercise- and study measurement related SAEs to the sponsor. As death, hospitalisation or persistent/significant disability or incapacity due to the disease itself may be expected in this study population, we will not collect these as SAEs.
The exercise- and study measurement related SAEs will be reported every twelve months (line-listings) to the Ethics Committee.

8.1.3. Follow-up of adverse events

(S)AEs need to be reported up to 30 days after the last study visit.

8.2. Data Safety Monitoring Board (DSMB)

Not applicable. We consider a DSMB not necessary since this is a low risk trial. The safety of physical exercise has been shown in previous studies in breast cancer survivors [25].

9. STATISTICAL ANALYSIS

Baseline demographics and characteristics will be reported by treatment group descriptively. Means and SDs will be presented for continuous normally distributed variables and medians and ranges for non-normally distributed variables. Categorical variables will be presented by frequencies per group (absolute numbers and percentages).

9.1. Primary study parameter(s)

Mixed models for repeated measures (MMRM) will be used to assess the intervention effects on physical fatigue and HRQoL, taking the hierarchical structure of the data into account (patients nested within centres). Independent factors will be time, group, and time * group, and cofactors. Cofactors include the baseline value of the outcome and the stratification factors (centre and therapy line). The primary contrast will be the comparison of the change from baseline to six months between groups. The statistical testing for the group effect will be done for each primary endpoint separately. The multiple test procedure will use a Bonferroni-Holm adjustment to maintain an overall alpha level of 5%.

In addition, potential moderators of the exercise effect will be investigated, e.g., age, baseline fitness level, type of therapy and type of metastases, by comparing the log likelihood of the model including the moderator variable with the log likelihood of the model including the moderator and its interaction term with the intervention.
To explore potential underlying mechanisms, mediation analyses will be performed for example for specific biomarkers.

Missing endpoint values due to disease-progression or mortality will occur, which could be Missing Not At Random (MNAR), e.g. if the exercise intervention would also have an effect on progression and survival. To explore potential bias, sensitivity analyses according to EMA recommendations will be conducted using approaches that investigate different MNAR scenarios such as a pattern mixture model.

9.2. Secondary study parameters

For all continuous secondary outcome measures (e.g. patient reported outcomes, fitness and body composition), we will use the same analytic approach as described above for the primary outcome measures.

To compare toxicity rates (Grade 3 and higher) between the intervention groups, we will graphically present the proportions per time point and will analyse the difference with a mixed model for binary data.

9.3. Exploratory analysis

To assess the potential effect of the intervention on progression-free, overall and breast-cancer specific survival, we will use Cox regression analyses stratified by centre and adjusted for pre-specified prognostic factors, i.e. therapy line, type of baseline metastases, age, time since diagnosis of first metastases. If factors appear highly correlated (e.g. time since diagnosis of first metastases and therapy line), we will chose one of the two.

9.4. Cost-effectiveness analysis

We will also perform an economic evaluation of the EFFECT exercise intervention for metastatic breast cancer patients. In this evaluation, the balance between costs and effects will be assessed. The net costs incurred with both strategies will be compared up until nine months after randomisation. Results of both cost and effect measurement will be integrated using cost-effectiveness and cost-utility analyses. In the cost-utility analysis, efficiency is expressed in terms of costs per Quality Adjusted Life Year. In the cost-effectiveness analysis, costs per unit of change in the two primary outcome measures (physical fatigue and overall HRQoL related symptom burden) will be estimated. Incremental costs and incremental effects, expressed in a ratio (ICER) will be estimated. To adjust for missing data, multiple imputation techniques will be used. Deterministic and probabilistic sensitivity analysis will be applied, using bootstrapping techniques. Results will be presented in a cost-effectiveness plane and a cost-effectiveness acceptability curve. The time horizon for the analysis will be nine months; hence, no discounting of costs and effects will be needed.

Additionally, country-specific analysis will be performed since local costs may vary importantly across countries. The relatively small patient groups available for these country-specific analyses imply that uncertainty will be larger than for the analysis at the overall level. Therefore, we will apply standard bootstrapping techniques to account for these uncertainties, using 5000 bootstraps as a minimum.

9.5. Other study parameters

Safety analyses will include tabulations of severe adverse events and chi-square tests.

Furthermore, adherence to the intervention, potential contamination of the control group due to high levels of physical activity, and number and reasons for drop outs will be analysed.

9.6. Interim analysis

Not applicable

10. ETHICAL CONSIDERATIONS

10.1. Regulation statement

The study will be conducted in full conformance with the principles of the "Declaration of Helsinki"(64th WMA General Assembly, Fortaleza, Brazil, October 2013) and with the laws and regulations of the country in which the research is conducted, whichever affords the greater protection to the participant. All study data will be handled confidentially. The researchers will handle all data complying with the General Data Protection Regulation (GDPR) (EU) 2016/679.

10.2. Regulatory Authority/ Independent Ethics Committees

The complete research file also including all materials provided to the participant (such as participant information sheets or descriptions of the study used to obtain informed consent as well as any recruitment materials or compensation given to the participant) will be submitted to the Regulatory Authorities and Ethics Committees in accordance with local procedures and regulatory requirements. Approval from the committee must be obtained before starting the study, and should be documented in a letter to the investigator specifying the date on which the committee met and granted the approval. Any modifications made to the protocol after receipt of the Regulatory Authorities / Ethics Committees approval must be re-submitted in accordance with local procedures and regulatory requirements.

10.3. Recruitment and consent

Patients will be informed about the EFFECT study either during a regular follow-up visit (oncologist or nurse) or via mail from their treating physician. Patients receive an information package including an invitation letter, a study leaflet and the patient information brochure. We will also use social media (e.g. of national/local patient organisations) to recruit patients. After a patient expresses interest through any type of contact, a member of the research staff will call the patient or meet with the

patient at the hospital. During the phone call/visit, the investigator or an authorised member of the study team explains the aims, methods, reasonably anticipated benefits, and potential burden of the study to the patient. Patients will be informed that their participation is voluntary and that they may withdraw consent to participate at any time. They will be informed that choosing not to participate will not affect the care they receive for the treatment of their disease. Furthermore, eligibility will be pre-screened.

Eligible and still interested patients will be invited for an informed consent visit which will be combined with the first measurements. The time interval between the first receipt of the patient information letter and the planned visit will be reasonable and at least one week so that patients have time to consider their decision.

During the consent visit, the above mentioned information on the study will be provided again. Thereafter, consent should be appropriately recorded by means of the participant's personally dated signature and authorised study staff's personally dated signature. After obtaining the consent, a copy of the ICF is given to the participant. The original will be maintained with the participant's records. If new safety information results in significant changes in the risk/benefit assessment, the ICF should be reviewed and updated if necessary. All participants should be informed of the new information and give their consent to continue the study.

10.4. Benefits and risks assessment, group relatedness

Burden and risk assessment

Study participation takes time, especially for patients in the intervention group.
Patients will visit the study centre (e.g. UMC Utrecht) for measurements at baseline, three months and six months. At nine months, patients will be asked to complete questionnaires at home.
Visits will take ~3 to 4 hours dependent on whether questionnaires will be completed at home or at the centre.
Patients in the <u>intervention group</u> are invited to participate in a 9-month exercise program involving two hours of supervised exercise per week (in addition to the encouragement to be active for at least 30 minutes on all remaining days of the week (5 days per week)). Burden of travelling to the training facilities will be reduced by offering the exercise program at centres close to the patients' home and replacing one supervised session to an unsupervised session after six months.

- Injuries due to exercise can occur; to minimize the risk, the intensity of the exercise program will be gradually increased during the study and supervised by a physiotherapist, exercise physiologist or qualified fitness instructor either face-to-face or live online.
- Every patient will be asked to wear an activity tracker (Fitbit) during the study.
- During the blood draws, a haematoma can occur after blood sampling.

- Incidental findings can arise during the different measurements (e.g. blood pressure measurement), which will be reported to participants and their treating physician when clinically relevant.

Overall, we consider that the risk of this study is marginal and the burden acceptable.

Benefit

We expect that the exercise program will have a beneficial effect on the patients' health status.

We believe that withholding the intervention under study from the control group is ethical for the following reasons:

- All control patients are offered an activity tracker (Fitbit) which is also a physical activity stimulus.
- Although exercise is a promising intervention for reducing fatigue in patients treated with curative intent, to date no clear evidence has been generated in patients with metastatic breast cancer, i.e. there is still clinical equipoise.

10.5. Compensation for injury

The sponsor has a liability insurance which is in accordance with Dutch legislation.

The sponsor has received dispensation from the statutory obligation to provide insurance for the subjects participating in the study, because the risk of participating in the study is low.

10.6. Incentives

Travel expenses for study centre visits for baseline and follow up assessments, at reasonable and fair terms, will be compensated. All patients may keep the activity tracker (Fitbit).

11. DATA COLLECTION, HANDLING AND RECORD RETENTION

11.1. Recording of Data

Study data will be captured using an eCRF (Castor®). Castor® is compliant with all relevant regulations, such as ICH E6 Good Clinical Practice and the General Data Protection Regulation (GDPR). Required data for this study are to be obtained from the participant's medical records/source documents or by direct entry, where the information was first recorded and then entered into the eCRF. Data from the eCRF will be encoded and stored in a study database. Only authorised site staff will be

allowed to enter data into the eCRF and make changes to eCRF data. Additionally, data will be obtained from online questionnaires filled in by the participants. Links to these questionnaires are sent to the email address of the participant. Email addresses are saved in an encrypted fashion, adding an extra safety to the storage of this sensitive information. Only authorised site staff will be allowed to see, add and adapt email addresses.In compliance with regulations, investigator(s) will maintain all eCRFs and all source documents supporting data collected from each participant, as well as all study documents. These records must be readily available for audit or inspection. Participant records or other source data must be kept for the maximum period of at least 15 years. If off-site archiving is used, all records should be retrieved and made available for review at the time of an audit or regulatory authority inspection. If it becomes necessary for the sponsor or the appropriate regulatory authority to review any documentation relating to this study, the Investigator must permit access to such reports.

11.2. Data Quality Assurance

The Investigator(s) will be responsible for ensuring eCRF data completeness and accuracy. The eCRFs will be reviewed by a monitor from the CRO for completeness and accuracy as described in the Monitoring Plan. Source document verification will be performed for a random sample. The eCRF data will also be reviewed internally by Data Management, medical and scientific staff and, if necessary, the investigational sites will be queried for corrections and/or clarifications. Once data are concluded to be complete and accurate, the eCRF data will be locked, meaning that the data will become read-only. The Investigator(s) are required to approve the eCRF data of their site through provisioning of an (electronic) signature before the data is used for final analysis. The sponsor will ensure that eCRF data is accessible and verifiable by the investigational site and install adequate back-up and security measures to prevent loss of data or unauthorised access to the data. Using the country specific guidelines and in coordination with the local PI, remote and/or on site monitoring will be applied. Remote source data verification (SDV) can be applied as mentioned in the local guidelines of the authorities. Remote SDV will focus on the quality control of critical data such as primary efficacy data and important safety data. Important secondary efficacy data may be monitored simultaneously if these are captured on the same documents. During remote SDV adequate data protection will be ensured, including data security and protection of personal data even if pseudonymised. The principal investigator (PI)/PI's institution and the sponsor will be jointly responsible as controllers for ensuring information is safeguarded. Details can be found in the monitor plan.

11.3. Data Management

The data-management of this multi-centre study will be conducted according to the data-management plan.

12.DEVIATION & VIOLATION

12.1. Deviation

A deviation is an accidental or unintentional change to, or non-compliance with, the research protocol that does not increase risk or decrease benefit or; does not have a significant effect on the participant's rights, safety or welfare and/or on the integrity of the data. Deviations may result from the action of the participant, investigator, or research staff.

12.2. Violation

A violation is an accidental or unintentional change to, or non-compliance with, the Ethics Committee approved protocol without prior sponsor and Ethics Committee approval. Violations generally increase risk or decrease benefit, affect the participant's rights, safety, or welfare, or the integrity of the data. A violation is also present in cases of wilful or knowing misconduct and in cases of serious or continuing non-compliance with laws and regulations.

12.3. Follow-up of Deviations and Violations

In case of either a deviation or a violation, the investigator enters a comment in the source documents and/or eCRF. The violations will also be documented in a Monitoring Visit Report by the Clinical Research Associate.
Violations affecting the safety of the participant will be escalated immediately to the sponsor. In parallel, corrective and/or preventive actions will be undertaken and documented, including any retraining of the investigator and site staff. All deviations and violations will be documented in the Trial Master File.

13. ADMINISTRATIVE ASPECTS, MONITORING AND PUBLICATION

13.1. Handling and storage of data and documents

The investigator must assure that the participants' anonymity will be maintained and that their identities are protected from unauthorised parties. In eCRFs, other documents and specimens submitted to the sponsor, participants should not be identified by their names, but by an identification code. The investigator should keep a participant enrolment log showing codes and names. The investigator should maintain documents not for submission to the sponsor, e.g. participants written informed consent forms, in strict confidence.
The data of the activity tracker will be extracted by our app (intervention group) and stored on local servers of our partner Nurogames in Germany. For privacy reasons, we will ask the patients not to use their name and last name in the app, but to use an acronym or nickname plus the studyID. For result evaluations, we will ask patients to provide their app credentials to the research team, so that we are able to extract the data. The tracker data of the control group will be extracted during the

study visits by using the credentials of the patient and saved at secured locations/servers at the study centres.

13.2. Monitoring and Quality Assurance

All source documents of the EFFECT study will be maintained by the Investigator(s) and made available for inspection by authorized persons including independent monitors, auditors, regulatory authorities and ethical committees where allowed by local law. The monitoring procedures of this study are described in the Project Reference Guide and the Monitoring Plan.

13.3. Amendments

Protocol and/or ICF modifications or changes may not be initiated without prior written Regulatory Authorities and Ethics Committee approval except when necessary to eliminate immediate hazards to the participants or when the change(s) involves only logistical or administrative aspects of the study. Such modifications will be submitted to the applicable Regulatory Authorities and Ethics Committee in accordance with local procedures and regulatory requirements. A written verification that the modification was submitted and subsequently approved should be obtained.

13.4. Annual progress report

The principal investigator will submit a summary of the progress of the trial to the applicable ethics committees once a year. Information will be provided on the date of inclusion of the first subject, numbers of subjects included and numbers of subjects that have completed the trial, serious adverse events/ serious adverse reactions potentially related to the study, other problems, and amendments.

13.5. Temporary halt and (prematurely) end of study report

The sponsor will notify the ethics committee of the end of the study within a period of 8 weeks. The end of the study is defined as the last patient’s last visit.

The sponsor will notify the ethics committee immediately of a temporary halt of the study, including the reason of such an action.

In case the study is ended prematurely, the sponsor will notify the ethics committee within 15 days, including the reasons for the premature termination.

13.6. Public disclosure and publication policy

The EFFECT study is registered in a public trial registry (ClinicalTrials.gov). We agree on the CCMO’s position on the disclosure/publication of the research results obtained from studies involving human subjects. The research data will be disclosed by the investigators on request after publication of the study results.

14.REFERENCES

(1) Harding V, Afshar M, Krell J, Ramaswami R, Twelves CJ, Stebbing J. 'Being there' for women with metastatic breast cancer: a pan-European patient survey. Br J Cancer 2013;109:1543-1548.

(2) Rainbird K, Perkins J, Sanson-Fisher R, Rolfe I, Anseline P. The needs of patients with advanced, incurable cancer. Br J Cancer 2009;101:759-764.

(3) Hofman M, Ryan JL, Figueroa-Moseley CD, Jean-Pierre P, Morrow GR. Cancer-related fatigue: the scale of the problem. Oncologist 2007;12 Suppl 1:4-10.

(4) Mustian KM, Alfano CM, Heckler C et al. Comparison of Pharmaceutical, Psychological, and Exercise Treatments for Cancer-Related Fatigue: A Meta-analysis. JAMA Oncol 2017;3:961-968.

(5) Baumann FT, Bloch W, Weissen A et al. Physical Activity in Breast Cancer Patients during Medical Treatment and in the Aftercare - a Review. Breast Care (Basel) 2013;8:330-334.

(6) van Vulpen JK, Peeters PH, Velthuis MJ, van der Wall E, May AM. Effects of physical exercise during adjuvant breast cancer treatment on physical and psychosocial dimensions of cancer-related fatigue: A meta-analysis. Maturitas 2016;85:104-111.

(7) Lipsett A, Barrett S, Haruna F, Mustian K, O'Donovan A. The impact of exercise during adjuvant radiotherapy for breast cancer on fatigue and quality of life: A systematic review and meta-analysis. Breast 2017;32:144-155.

(8) Juvet LK, Thune I, Elvsaas IKO et al. The effect of exercise on fatigue and physical functioning in breast cancer patients during and after treatment and at 6 months follow-up: A meta-analysis. Breast 2017;33:166-177.

(9) Courneya KS, Segal RJ, Mackey JR et al. Effects of aerobic and resistance exercise in breast cancer patients receiving adjuvant chemotherapy: a multicenter randomized controlled trial. J Clin Oncol 2007;25:4396-4404.

(10) van Waard H, Stuiver MM, van Harten WH et al. Effect of Low-Intensity Physical Activity and Moderate- to High-Intensity Physical Exercise During Adjuvant Chemotherapy on Physical Fitness, Fatigue, and Chemotherapy Completion Rates: Results of the PACES Randomized Clinical Trial. J Clin Oncol 2015;33:1918-1927.

(11) Mijwel S, Backman M, Bolam KA et al. Adding high-intensity interval training to conventional training modalities: optimizing health-related outcomes during chemotherapy for breast cancer: the OptiTrain randomized controlled trial. Breast Cancer Res Treat 2018;168:79-93.

(12) Schmidt ME, Wiskemann J, Armbrust P, Schneeweiss A, Ulrich CM, Steindorf K. Effects of resistance exercise on fatigue and quality of life in breast cancer patients undergoing adjuvant chemotherapy: A randomized controlled trial. Int J Cancer 2015;137:471-480.

(13) Steindorf K, Schmidt ME, Klassen O et al. Randomized, controlled trial of resistance training in breast cancer patients receiving adjuvant radiotherapy: results on cancer-related fatigue and quality of life. Ann Oncol 2014;25:2237-2243.

(14) Travier N, Velthuis MJ, Steins Bisschop CN et al. Effects of an 18-week exercise programme started early during breast cancer treatment: a randomised controlled trial. BMC Med 2015;13:121.

(15) Steins Bisschop CN, Courneya KS, Velthuis MJ et al. Control group design, contamination and drop-out in exercise oncology trials: a systematic review. PLoS One 2015;10:e0120996.

(16) Weis J, Tomaszewski KA, Hammerlid E et al. International Psychometric Validation of an EORTC Quality of Life Module Measuring Cancer Related Fatigue (EORTC QLQ-FA12). J Natl Cancer Inst 2017;109.

(17) Giesinger JM, Kieffer JM, Fayers PM et al. Replication and validation of higher order models demonstrated that a summary score for the EORTC QLQ-C30 is robust. J Clin Epidemiol 2016;69:79-88.

(18) Kyle UG, Genton L, Karsegard L, Slosman DO, Pichard C. Single prediction equation for bioelectrical impedance analysis in adults aged 20--94 years. Nutrition 2001;17:248-253.

(19) De Backer IC, Schep G, Hoogeveen A, Vreugdenhil G, Kester AD, van BE. Exercise testing and training in a cancer rehabilitation program: the advantage of the steep ramp test. Arch Phys Med Rehabil 2007;88:610-616.

(20) Stuiver MM, Kampshoff CS, Persoon S et al. Validation and Refinement of Prediction Models to Estimate Exercise Capacity in Cancer Survivors Using the Steep Ramp Test. Arch Phys Med Rehabil 2017;98:2167-2173.

(21) Guralnik JM, Simonsick EM, Ferrucci L et al. A short physical performance battery assessing lower extremity function: association with self-reported disability and prediction of mortality and nursing home admission. J Gerontol 1994;49:M85-M94.

(22) Amireault S, Godin G. The Godin-Shephard leisure-time physical activity questionnaire: validity evidence supporting its use for classifying healthy adults into active and insufficiently active categories. Percept Mot Skills 2015;120:604-622.

(23) Amireault S, Godin G, Lacombe J, Sabiston CM. Validation of the Godin-Shephard Leisure-Time Physical Activity Questionnaire classification coding system using accelerometer assessment among breast cancer survivors. J Cancer Surviv 2015;9:532-540.

(24) Amireault S, Godin G, Lacombe J, Sabiston CM. The use of the Godin-Shephard Leisure-Time Physical Activity Questionnaire in oncology research: a systematic review. BMC Med Res Methodol 2015;15:60.

(25) Fong DY, Ho JW, Hui BP et al. Physical activity for cancer survivors: meta-analysis of randomised controlled trials. BMJ 2012;344:e70.

(26) de Jong, M. (2016). Quality of Working Life of cancer survivors: Development and evaluation of a measurement instrument https://bmccancer.biomedcentral.com/track/pdf/10.1186/s12885-017-3966-1.

(27) https://www.eortc.be/itemlibrary/

(28) Csuka M, McCarty DJ. Simple method for measurement of lower extremity muscle strength. Am J Med. 1985 Jan;78(1):77-81 PMID 3966492

(29) Guralnik, J. M., Simonsick, E. M., Ferrucci, L., Glynn, R. J., Berkman, L. F., Blazer, D. G., ... & Wallace, R. B. (1994). A short physical performance battery assessing lower extremity function: association with self-reported disability and prediction of mortality and nursing home admission. Journal of gerontology, 49(2), M85-M94.

(30) Rose DJ1, Lucchese N, Wiersma LD. Development of a multidimensional balance scale for use with functionally independent older adults. Arch Phys Med Rehabil. 2006 Nov;87(11):1478-85.

(31) Wampler MA1, Topp KS, Miaskowski C, Byl NN, Rugo HS, Hamel K. Quantitative and clinical description of postural instability in women with breast cancer treated with taxane chemotherapy. Arch Phys Med Rehabil. 2007 Aug;88(8):1002-8.

(32) Avery K, Donovan J, Peters T, Shaw C, Gotoh M, & Abrams P. ICIQ: a brief and robust measure for evaluating the symptoms and impact of urinary incontinence. Neurourol.Urodyn. 2004; 23(4):322-30.

APPENDIX 1. Detailed description of the EFFECT exercise program

Balance and Core Training<br>*(for adaption due to bone met., see Tab. 1)*							
**Duration (min)**		**Exercises**				**Intensity**	**Target Body Region**
**overall**	**single**						
5		***Initial exercises (progressing from left to right)***					
		Step 1	Step 2	Step 3	Step 4		
		Side-by-side stand	Tandem stand	Heel to toe stand	Feet apart in line stand		
		Normal walk	Sideways walk	Heel to toe walk	Grape vine		
		More challenging exercises (progressing from left to right)					
		Step 1	Step 2	Step 3	Step 4		
		1 leg stand	1 leg stand eyes closed	1 leg stand with upper arm exercise	1 leg stand with upper arm exercise and trunk rotation		
		Normal Backwards walk	Backwards walk on line	Heel to toe backwards walk	Heel to toe walk with turn around		
		Dual tasking stand	Dual tasking with squat	Dual tasking forwards walk	Dual tasking sideways walk		
		Gluteal bridge	Gluteal bridge + alternating heel raise	Gluteal bridge + alternating bent leg raise	Gluteal bridge + alternating stretched leg raise		
		All fours + alternating arm raise	All fours + alternating arm + leg raise	All fours + alternating arm + leg raise (arm/leg stretched)	All fours + alternating arm + leg raise (arm/leg stretched) + approach arm/leg		

Resistance Training<br>*(for adaption due to bone met., see Tab. 1)*												
**Duration (min)**		**Exercises**			**Intensity**							**Target Body Region**
**overall**	**single**											
		**Variation 1 (machine-based)**	**Variation 2 (free weights)**	**Variation 3 (body weight)**	Week 1-4	Week 5-8	Week 9-12	Week 13-16	Week 17-20	…	Week 33-36	
					1 min rest between sets							
**35**	6	**Leg press**	Squad (with weights) **or** Lunge	Squad **or** Lunge **or** Sit-to-Stand (without weights)	10-12 reps, 3 sets	6-8 reps, 3 sets	10-12 reps, 3 sets	6-8 reps, 3 sets	10-12 reps, 3 sets	…	10-12 reps, 3 sets	lower body
	6	**Leg curl**	Prone leg curl with ankle weights	Step ups **or** lunge Supine bridge	10-12 reps, 3 sets	6-8 reps, 3 sets	10-12 reps, 3 sets	6-8 reps, 3 sets	10-12 reps, 3 sets	…	10-12 reps, 3 sets	lower body
	6	**Leg extension**	Leg extension with ankle weights	Step ups **or** lunge Supine bridge	10-12 reps, 3 sets	6-8 reps, 3 sets	10-12 reps, 3 sets	6-8 reps, 3 sets	10-12 reps, 3 sets	…	10-12 reps, 3 sets	lower body
	6	**Chest press**	Dumbbell bench press on bench/floor **or** Forward pushing of medicine ball (lying position)	Low level push ups	10-12 reps, 3 sets	6-8 reps, 3 sets	10-12 reps, 3 sets	6-8 reps, 3 sets	10-12 reps, 3 sets	…	10-12 reps, 3 sets	upper body
	6	**Seated row machine**	Dumbbell rowing (1 vs 2 arms) **or** T-Bar, if available **or** Thera-Band (pull from front to the back	Seated row with resistance bands tied around post/pole/door (could also be performed standing)	10-12 reps, 3 sets	6-8 reps, 3 sets	10-12 reps, 3 sets	6-8 reps, 3 sets	10-12 reps, 3 sets	…	10-12 reps, 3 sets	upper body
	6	**Lat pulldown**	Thera-Band (overhead fixing and pull down) **or** pull-overs with dumbbells	Pull ups with resistance band(s)	10-12 reps, 3 sets	6-8 reps, 3 sets	10-12 reps, 3 sets	6-8 reps, 3 sets	10-12 reps, 3 sets	…	10-12 reps, 3 sets	upper body
	**FURTHER OPTIONAL EXERCISES**											

	6	Calf raises	Calf raises with dumbbells	Calf raises without any modification **or** at stairs	10-12 reps, 3 sets	6-8 reps, 3 sets	10-12 reps, 3 sets	6-8 reps, 3 sets	10-12 reps, 3 sets	…	10-12 reps, 3 sets	lower body
	6	Shoulder press	Overhead lifting of dumb-bells (sitting or standing)	Frontal/lateral raise	10-12 reps, 3 sets	6-8 reps, 3 sets	10-12 reps, 3 sets	6-8 reps, 3 sets	10-12 reps, 3 sets	…	10-12 reps, 3 sets	upper body
	6	Butterfly	Thera-Band (pull from back to the front)	Low level push ups	10-12 reps, 3 sets	6-8 reps, 3 sets	10-12 reps, 3 sets	6-8 reps, 3 sets	10-12 reps, 3 sets	…	10-12 reps, 3 sets	upper body
	6	Back extensor	Deadlift with no or low weight	Chair back extension	10-12 reps, 3 sets	6-8 reps, 3 sets	10-12 reps, 3 sets	6-8 reps, 3 sets	10-12 reps, 3 sets	…	10-12 reps, 3 sets	upper body
	6	Ab Core (ab-dominal) ma-chine	Lifting of one leg with bent knee while standing (with weight cuffs); improvement: lifting with straightened leg	Lifting of straight-ened leg while standing (without weight cuffs) **or** Plank	10-12 reps, 3 sets	6-8 reps, 3 sets	10-12 reps, 3 sets	6-8 reps, 3 sets	10-12 reps, 3 sets	…	10-12 reps, 3 sets	upper body
	6	Triceps exten-sions	Triceps extensions with dumbbells	Triceps dips on bench	10-12 reps, 3 sets	6-8 reps, 3 sets	10-12 reps, 3 sets	6-8 reps, 3 sets	10-12 reps, 3 sets	…	10-12 reps, 3 sets	upper body
	6	Biceps curls	Biceps curls with dumbbells	Bicep curl with re-sistance bands	10-12 reps, 3 sets	6-8 reps, 3 sets	10-12 reps, 3 sets	6-8 reps, 3 sets	10-12 reps, 3 sets	…	10-12 reps, 3 sets	upper body

Aerobic Training

(for adaption due to bone met., see Tab. 1)

Duration (min) overall	Duration (min) single	Exercises	Intensity: Week 1-3	Intensity: Week 4-14	Intensity: Week 15-25	Intensity: Week 26-36	Target Body Region
**15**		Cycle ergometer	15 min MICT (Start: 50% MSEC Goal: 60% MSEC)	Interval (8x1 min with 1 min rest between inter-vals)	Interval (3x3 min with 2 min rest between inter-vals)	Interval (8x30sec with 1 min rest between inter-vals)	Lower Body
		Elliptical/Cross trainer	15 min MICT (Start: 50% MSEC Goal: 60% MSEC)	Interval (8x1 min with 1 min rest between inter-vals)	Interval (3x3 min with 2 min rest between inter-vals)	Interval (8x 30sec with 1 min rest between intervals)	Lower Body
		Rowing machine	15 min MICT (Start: 50% MSEC Goal: 60% MSEC)	Interval (8x1 min with 1 min rest between inter-vals)	Interval (3x3 min with 2 min rest between inter-vals)	Interval (8x 30sec with 1 min rest between intervals)	Whole Body
		Treadmill	15 min MICT (Start: 50% MSEC Goal: 60% MSEC)	Interval (8x1 min with 1 min rest between inter-vals)	Interval (3x3 min with 2 min rest between inter-vals)	Interval (8x 30sec with 1 min rest between intervals)	WholeBody/ Lower Body

Cool down

Duration (min) overall	Duration (min) single	Exercises	Target Body Region
**5**		STRETCHING	Whole body

[1]Individual technical introduction into muscle group specific exercises (machine-based, free weights, body weight) by the supervising therapist during the first weeks. If necessary, the supervising therapist prepares the alternative exercises before training. During the 9-months intervention period, the weights are continuously adjusted according to a progressive training protocol and in line with the periodization (if it is consistent with the participant´s health status) so that the predefined maximum number of repetitions will not be exceeded. The participants will conduct the exercises according to the agonist-antagonist principle.

[2]During the first sessions: the therapist explains the App for mobile and tablet devices to the patient. In the course of the trial: the therapist is updated on the patient´s progress of prescribed weekly activity.

Table 1: Adaption of exercise program according to location of metastases

Metastases Site	Resistance Exercise			Aerobic Exercise		Flexibility
	Upper	Trunk	Lower	WB	NWB	Static
**Pelvis**	√	√	√**		√	√
**Axial Skeleton (lumbar)**	√		√		√	√***
**Axial Skeleton (thoracic/ribs)**	√*		√	√	√	√***
**Proximal Humerus**		√*	√	√	√	√*
**Proximal Femur**	√	√	√**		√	√
**All regions**	√*		√**		√	√***

√ = Target exercise region, * = exclusion of shoulder flexion/extension /abduction/adduction & inclusion of elbow flexion/extension, ** = exclusion of hip extension/flexion & inclusion of knee extension/flexion, *** = exclusion of spine/flexion/extension/rotation, WB = weight bearing (e.g. walking), NWB = non-weight bearing (e.g. cycling)

LITERATURE:

Wind, A.E. et al. (2010). Is grip strength a predictor for total muscle strength in healthy children, adolescents, and young adults? Eur J Pediatr 169:281–287
Landers J. Maximums based on reps. Natl Strength Cond Assoc J 1985; 6: 60–61
Courneya KS, Segal RJ, Mackey JR, Gelmon K, Reid RD, Friedenreich CM, Ladha AB, Proulx C, Vallance JK, Lane K, Yasui Y, McKenzie DC. Effects of aerobic and resistance exercise in breast cancer patients receiving adjuvant chemotherapy: a multicenter randomized controlled trial. J Clin Oncol 2007; 25: 4396– 4404

APPENDIX 2: Example of the training documentation of the supervised exercise Session

ID:

TRAINING ASSESSMENT FORM

Week 1 – Session 1	
PRE-TRAINING ASSESSMENT	**Last session**
Since your last session, did any injuries, problems, or symptoms occur during or after physical exertion or exercise...	
... that required treatment, hospitalization, or talking to a doctor or other health professional?	☐ no ☐ yes*
... that caused any other persistent worsening of your health or well-being (e.g. muscle strains, new occurrence of persistent pain or swelling)?	☐ no ☐ yes*
... that led to an increased intake of medication or prescription of new medication due to bone pain?	☐ no ☐ yes*

	**Balance Training**		**Resistance Training (repetitions: 10-12) | 35min**							**Endurance Training (MICT) | 15min**							
**Session**	**Resting HR**	**Balance or Core**	**Exercises**	**Repetitions** Set 1	Set 2	Set 3	**Weight in kg**	**RPE**	**Adjusted due to complications**	**Cardio device**	**Completed intervals**	**Watts or distance**	**RPE**	**Adjusted due to complications**	**Ø HR**	**Ø RPE**	Date & trainer Initials
**1**<br>Attendance<br>☐ yes<br>☐ no<br>If no:<br>☐ Sick/injured<br>☐ Other commitment<br>☐ Holiday			1.						☐ no<br>☐ yes<br>Adjustments:	Cycling ☐<br>Elliptical/ cross ☐<br>Rowing ☐<br>Treadmill ☐				☐ no<br>☐ yes<br>Adjustments:			(date)<br>(sign)
			2.														
			3.														
			4.														
			5.														
			6.														

POST-TRAINING ASSESSMENT	**Current session**
Did the training cause or aggravate any pain?	☐ no ☐ mild ☐ moderate ☐ substantial increase in pain*
If **moderate/substantial**, please describe:	
Did any other symptoms/problems occur or worsen during the session?	☐ no ☐ yes*
If **yes**, please describe:	
Did any problems/symptoms occur during the session that required restrictions/alterations or early termination of the training?	☐ no ☐ yes*

*If yes, please notify the Study Coordinator immediately: **<Site Specific Phone Number>** and use Safety Reporting Form for discussion/background information.

Comments:____

Supplementary Table 1. Self-reported and measured levels of physical activity, presented as median (IQR).

		**Baseline**	**3 months**	**6 months**	**9 months**
**Self-reported physical activity levels using the Godin-Shephard Leisure-Time Exercise Questionnaire** (median (IQR))					
Aerobic exercise					
Vigorous intensity (min/week)	EX<br>UC	0 (0-0)<br>0 (0-0)	30 (0-40)<br>0 (0-0)	30 (1-43)<br>0 (0-0)	12 (0-30)<br>0 (0-0)
Moderate intensity (min/week)	EX<br>UC	0 (0-58)<br>0 (0-60)	40 (1-120)<br>23 (0-90)	42 (0-100)<br>43 (0-135)	40 (0-90)<br>0 (0-60)
Light intensity (min/week)	EX<br>UC	30 (0-150)<br>15 (0-132)	50 (0-130)<br>60 (0-150)	60 (0-150)<br>60 (0-139)	31 (0-100)<br>40 (0-150)
Resistance exercise (min/week)	EX<br>UC	0 (0-0)<br>0 (0-0)	60 (15-90)<br>0 (0-20)	60 (1-80)<br>0 (0-1)	30 (0-50)<br>0 (0-15)
**Measured physical activity using the Fitbit Inspire HR** (median (IQR))					
Steps/day	EX<br>UC	7279 (4969 – 9665)<br>8240 (4791 – 11480)	7735 (5632 – 10147)<br>7249 (3751 – 11290)	7454 (4613 – 10841)<br>6368 (3448 – 10220)	7507 (4107 – 10521)<br>6864 (3277 – 10430)
Sedentary (min/day)	EX<br>UC	744 (627-958)<br>688 (607-879)	685 (610-876)<br>684 (570-897)	695 (618-842)<br>705 (604-933)	702 (599-864)<br>724 (605-1017)
Lightly Active (min/day)	EX<br>UC	220 (184-271)<br>235 (173-295)	245 (192-293)<br>238 (171-297)	222 (184-280)<br>221 (179-290)	236 (177-277)<br>210 (146-292)
Fairly Active (min/day)	EX<br>UC	15 (7-26)<br>16 (8-26)	15 (10-25)<br>12 (5-28)	12 (6-26)<br>14 (3-26)	12 (5-28)<br>11 (8-20)
Very Active (min/day)	EX<br>UC	14 (7-28)<br>17 (4-31)	14 (9-23)<br>10 (3-29)	13 (4-29)<br>6 (1-18)	13 (4-31)<br>10 (3-18)

Supplementary Figure 1. Screenshots of the PREFERABLE exercise App.

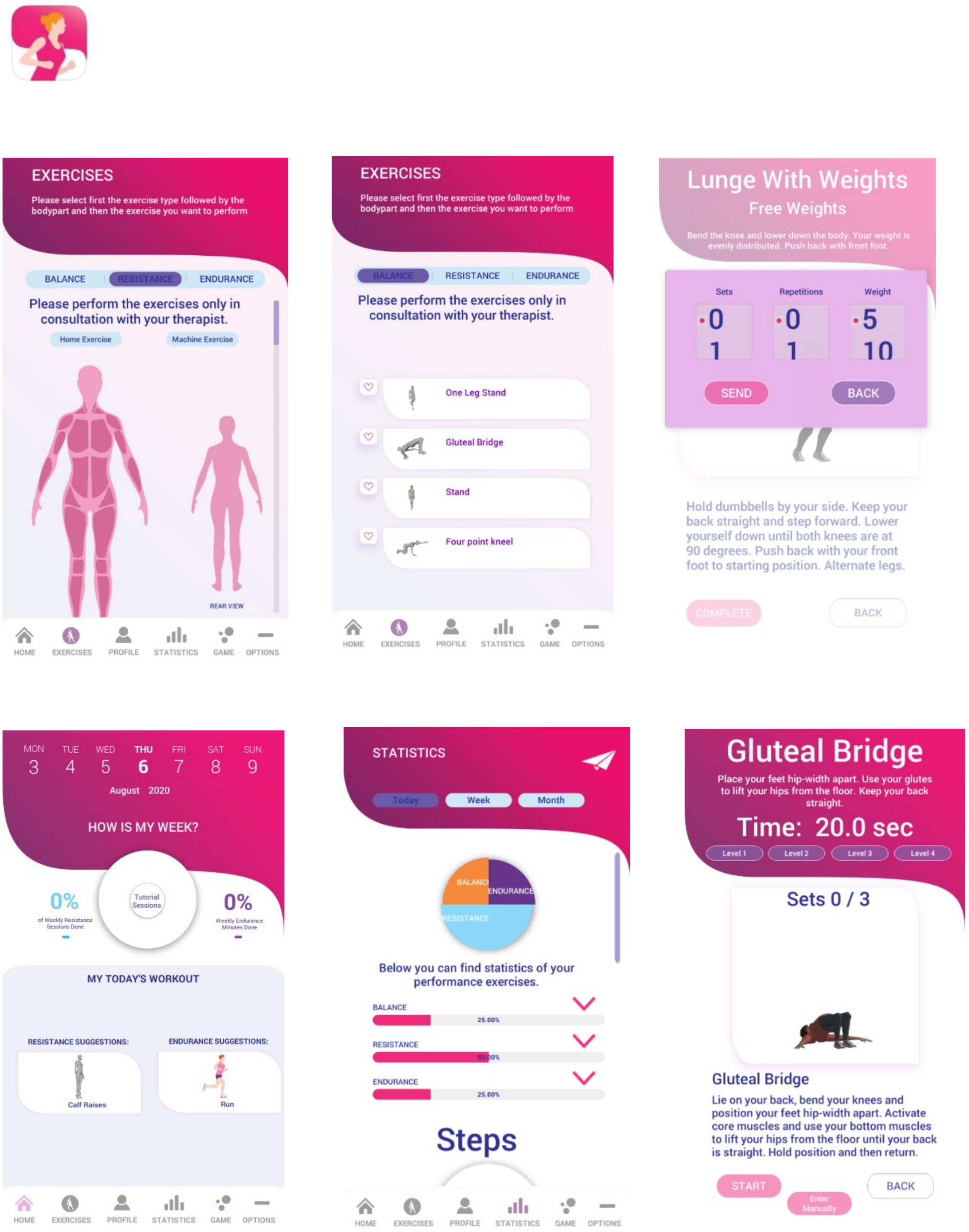

Supplementary Table 2. ***Additional physical functioning items from the EORTC item bank.***

	Not at all	A little	Quite a bit	Very much
Do you have any trouble hiking 3 km/2 mi on uneven surfaces?				
Do you have any trouble running fast?				
Do you have any trouble carrying a heavy bag upstairs?				
Do you have any trouble running a short distance, such as to catch the bus?				
