# Supplementary material for: Supervised, structured and individualized exercise in metastatic breast cancer: a randomized controlled trial
Source: Nat Med. 2024 Jul 25;30(10):2957–66. doi: 10.1038/s41591-024-03143-y (PMC11485212; doi:10.1038/s41591-024-03143-y)
Supplement: Supplementary file 2 — Reporting Summary [file 41591_2024_3143_MOESM2_ESM.pdf]

nature portfolio

Corresponding author(s): Prof. dr. Anne May

Last updated by author(s): Jun 18, 2024

Reporting Summary

Nature Portfolio wishes to improve the reproducibility of the work that we publish. This form provides structure for consistency and transparency in reporting. For further information on Nature Portfolio policies, see our Editorial Policies and the Editorial Policy Checklist.

Statistics

For all statistical analyses, confirm that the following items are present in the figure legend, table legend, main text, or Methods section.

n/a	Confirmed	
☐	☒	The exact sample size (*n*) for each experimental group/condition, given as a discrete number and unit of measurement
☐	☒	A statement on whether measurements were taken from distinct samples or whether the same sample was measured repeatedly
☐	☒	The statistical test(s) used AND whether they are one- or two-sided<br>*Only common tests should be described solely by name; describe more complex techniques in the Methods section.*
☐	☒	A description of all covariates tested
☐	☒	A description of any assumptions or corrections, such as tests of normality and adjustment for multiple comparisons
☐	☒	A full description of the statistical parameters including central tendency (e.g. means) or other basic estimates (e.g. regression coefficient) AND variation (e.g. standard deviation) or associated estimates of uncertainty (e.g. confidence intervals)
☐	☒	For null hypothesis testing, the test statistic (e.g. *F*, *t*, *r*) with confidence intervals, effect sizes, degrees of freedom and *P* value noted<br>*Give P values as exact values whenever suitable.*
☒	☐	For Bayesian analysis, information on the choice of priors and Markov chain Monte Carlo settings
☒	☐	For hierarchical and complex designs, identification of the appropriate level for tests and full reporting of outcomes
☐	☒	Estimates of effect sizes (e.g. Cohen's *d*, Pearson's *r*), indicating how they were calculated

Our web collection on statistics for biologists contains articles on many of the points above.

Software and code

Policy information about availability of computer code

Data collection	No specific software was used for data collection, except for physical activity data, which was captured using a Fitbit Inspire HR. Collected data were entered using the Castor electronic data collection system.
Data analysis	R version 4.2.2 has been used for data analysis. The script will be made available before publication via Github: https://github.com/AnoukHiensch/PREFERABLE-EFFECT.git

For manuscripts utilizing custom algorithms or software that are central to the research but not yet described in published literature, software must be made available to editors and reviewers. We strongly encourage code deposition in a community repository (e.g. GitHub). See the Nature Portfolio guidelines for submitting code & software for further information.

Data

Policy information about availability of data

All manuscripts must include a data availability statement. This statement should provide the following information, where applicable:

- Accession codes, unique identifiers, or web links for publicly available datasets
- A description of any restrictions on data availability
- For clinical datasets or third party data, please ensure that the statement adheres to our policy

The data that support the findings of this study are not yet openly available due to reasons of confidentiality. Researchers can request current data from the corresponding author (a.m.may@umcutrecht.nl). Pseudonymized data (including data dictionaries) will be made available via the Digital Research Environment

(DRE), which is a trusted digital research environment and can be accessed via https://mydre.org. This will be done after review and approval of a methodologically sound proposal by the General Assembly of PREFERABLE, with a signed data access agreement, in line with Ethics Committee requirements. Requests will be processed within 6 weeks. These files will be available from the date of publication until the date stated in the approved request. Once the PREFERABLE project has been fully completed, the database will be anonymized and shared via DataverseNL. The study protocol is available as an open access publication (doi: 10.1186/s13063-022-06556-7).

Research involving human participants, their data, or biological material

Policy information about studies with human participants or human data. See also policy information about sex, gender (identity/presentation), and sexual orientation and race, ethnicity and racism.

Reporting on sex and gender	Patients were enrolled regardless of sex, which was collected according to the identity information provided by the patients. As indicated by the journal, gender-based analyses are discouraged, if the study design is insufficient (for example low sample size) to enable meaningful conclusions, as is the case here. We only included two male participants and therefore sample sizes are too small to report disaggregated data.
Reporting on race, ethnicity, or other socially relevant groupings	No information on race, ethnicity or other socially relevant groupings was collected.
Population characteristics	In total, 856 patients were invited to take part in the PREFERABLE-EFFECT study. Of these, 357 provided consent and were enrolled (recruitment rate, 41.7%), with 178 randomized to the exercise group and 179 to the control group. The exercise and control groups had similar sociodemographic and clinical characteristics at baseline. The mean age of the participants was 55.4 years (SD=11.1), the majority was female (99.4%), was receiving 1st or 2nd line of treatment (74.8%) and had bone metastases (67.2%).
Recruitment	Potentially eligible patients were informed about the study by an oncology nurse or medical specialist during a regular visit or by mail/letter. In addition, social media (e.g., of national/local patient organizations) was used to recruit patients. Interested patients received an informational letter explaining the study aims and procedures. After 1 week, the patient was approached by telephone to provide further information, answer questions and check the (remaining) in- and exclusion criteria. Eligible patients who were willing to participate were invited to the study center to sign written informed consent and to undergo baseline measurements.<br>Due to (self-)selection by the patient and/or treating physician, patients who were enrolled might have had certain characteristics (e.g., completed higher education) that made them more prone to participate, while patients in greatest need of an exercise intervention (i.e., with higher fatigue levels, more co-morbidities) might have been less likely to participate. This could have resulted in an underestimation of the intervention effect.
Ethics oversight	The study was approved by the institutional review board of the University Medical Center Utrecht, the Netherlands (19-524/M), and by the local ethical review boards of all participating institutions: (Heidelberg University Hospital/German Cancer Research Center (DKFZ)/National Center for Tumor Diseases (NCT) Heidelberg and German Sport University Cologne (DSHS)), the Netherlands (the Netherlands Cancer Institute (NKI)), Poland (Wielkopolskie Centrum Onkologii (WCO) and Greater Poland Cancer Center), Spain (Onkologikoa (ONK)), Sweden (Karolinska Institutet (KI)), and Australia (Australian Catholic University (ACU)).

Note that full information on the approval of the study protocol must also be provided in the manuscript.

Field-specific reporting

Please select the one below that is the best fit for your research. If you are not sure, read the appropriate sections before making your selection.

☒ Life sciences ☐ Behavioural & social sciences ☐ Ecological, evolutionary & environmental sciences

For a reference copy of the document with all sections, see nature.com/documents/nr-reporting-summary-flat.pdf

Life sciences study design

All studies must disclose on these points even when the disclosure is negative.

Sample size	An improvement on either or both primary outcomes in the exercise group from baseline to 6 months post-baseline relative to the control group was of primary relevance. Based on a pooled analysis of 6 randomized controlled exercise trials in patients with breast cancer receiving adjuvant treatment, we anticipated an effect size of 0.35. With n=139 patients per group (n=278 in total), for each outcome separately a mean standardized effect size of at least 0.35 could be detected with an analysis of covariance (ANCOVA) adjusted for baseline values of the outcome variable with a power of at least 78% or 82% at a (nominal) two-sided significance level of 2.5%, assuming a correlation between pre- and post-intervention levels of Rho=0.3 or Rho=0.4, respectively.26 To account for a potential drop-out rate of approximately 20%, the target sample size was 350 participants (n = 175 per study arm).
Data exclusions	Data was analyzed according to the intention-to-treat principle and the analyses included participants for whom the outcome was observed at two or more time points.
Replication	A statistical analysis plan was written before the analysis was performed and included in the study protocol. The main analyses have been performed by three independent researchers to check reproducibility, which could be confirmed.
Randomization	Patients who met the eligibility criteria and provided informed consent were randomly assigned (1:1), after completion of the baseline

Randomization	measurements, to receive a 9-month structured and individualized exercise program in addition to usual care (hereafter referred to as 'the exercise group') or general physical activity advice in addition to usual care, but no structured exercise program (hereafter referred to as 'the control group'). All participants received an activity tracker.<br>Randomisation was performed centrally using a blocked computer-generated sequence and was stratified by study centre and therapy line (1st or 2nd line treatment vs 3rd or later line).
Blinding	Due to the nature of the intervention, participants, local clinicians and study nurses, and investigators were not blinded to group assignment after randomisation.

Reporting for specific materials, systems and methods

We require information from authors about some types of materials, experimental systems and methods used in many studies. Here, indicate whether each material, system or method listed is relevant to your study. If you are not sure if a list item applies to your research, read the appropriate section before selecting a response.

Materials & experimental systems

n/a	Involved in the study	
☒	☐	Antibodies
☒	☐	Eukaryotic cell lines
☒	☐	Palaeontology and archaeology
☒	☐	Animals and other organisms
☐	☒	Clinical data
☒	☐	Dual use research of concern
☒	☐	Plants

Methods

n/a	Involved in the study	
☒	☐	ChIP-seq
☒	☐	Flow cytometry
☒	☐	MRI-based neuroimaging

Clinical data

Policy information about clinical studies
All manuscripts should comply with the ICMJE guidelines for publication of clinical research and a completed CONSORT checklist must be included with all submissions.

Clinical trial registration	This study is registered with ClinicalTrials.gov, NCT04120298
Study protocol	The study protocol is available as an open access publication (doi: 10.1186/s13063-022-06556-7) and has been added as supplement to this publication.
Data collection	The PREFERABLE-EFFECT multinational randomised controlled trial (RCT) was undertaken at eight hospitals/study centres in Germany, the Netherlands, Poland, Spain, Sweden, and Australia. Between January 8, 2020 and August 3, 2022, a total of 856 patients were invited to take part in the PREFERABLE-EFFECT study. Of these, 357 provided consent and were enrolled (recruitment rate, 41.7%), with 178 randomized to the exercise group and 179 to the control group.
Outcomes	The study had two primary outcomes: health-related quality of life (HRQoL) and cancer-related physical fatigue, assessed at 6 months using the summary score of the QLQ-C30 and the physical fatigue dimension of the QLQ-FA12, respectively.<br>Secondary outcomes reported in this paper include the primary outcomes assessed at 3- and 9-months, as well as a range of other variables: the QLQ-C30 global QoL score, and all other QLQ-C30 function and symptom scales and single items, all other QLQ-FA12 fatigue dimensions, and the MSEC.

Plants

Seed stocks	*Report on the source of all seed stocks or other plant material used. If applicable, state the seed stock centre and catalogue number. If plant specimens were collected from the field, describe the collection location, date and sampling procedures.*
Novel plant genotypes	*Describe the methods by which all novel plant genotypes were produced. This includes those generated by transgenic approaches, gene editing, chemical/radiation-based mutagenesis and hybridization. For transgenic lines, describe the transformation method, the number of independent lines analyzed and the generation upon which experiments were performed. For gene-edited lines, describe the editor used, the endogenous sequence targeted for editing, the targeting guide RNA sequence (if applicable) and how the editor was applied.*
Authentication	*Describe any authentication procedures for each seed stock used or novel genotype generated. Describe any experiments used to assess the effect of a mutation and, where applicable, how potential secondary effects (e.g. second site T-DNA insertions, mosiacism, off-target gene editing) were examined.*
